# Transformation Behavior of Fe, V and Ti in Pre–Reduced Vanadium–Titanium Magnetite During Electric Furnace Smelting

**DOI:** 10.3390/ma19214582

**Published:** 2026-10-29

**Authors:** Conglin Yao, Baolin Tan, Bing Hu, Fuqiang Zheng, Yong Yu, Pengbo Wang

**Affiliations:** 1 Zhongye Changtian International Engineering Co., Ltd., Changsha 410205, China; 2 Engineering Research Center of Metallurgical Energy Conservation and Emission Reduction, Ministry of Education, School of Metallurgical and Energy Engineering, Kunming University of Science and Technology, Kunming 650093, China; 3 Department of Industrial and Systems Engineering, The Hong Kong Polytechnic University, Hong Kong, China

**Keywords:** vanadium–titanium magnetite, transformation mechanisms of Fe, V and Ti, direct reduction–electric furnace process, efficient resource utilization

## Abstract

**Highlights:**

- The migrations of Fe, V and Ti during reduction smelting were clarified.
- The recovery ratios of V, Fe and Ti reached 94.56%, 99.61% and 98.87%.
- This paper has significant implications for pre–reduced vanadium–titanium magnetite smelting.

**Abstract:**

Vanadium–titanium magnetite is an important global strategic resource, with proven reserves exceeding 40 billion tons. Currently, the direct reduction–electric furnace route is regarded as the most promising process for VTM treatment. However, the transformation mechanisms of Fe, V and Ti remain insufficiently understood, constraining industrial implementation. In this study, the transformations of Fe, V and Ti during reduction smelting were systematically clarified, and the effects of MgO, CaO and semi–coke were evaluated. MgO addition promoted the formation of MgAl_2_O_4_, (Ti,V)_2_O_3_ and (Mg,V)_x_Ti_3–x_O_5_, hindering slag–alloy separation. In contrast, with CaO as a modifier, FeTiO_3_ in the raw material was reduced by C to yield Fe and V_x_Ti_3–x_O_5_, which was further reduced to (Ti,V)_2_O_3_. The added CaO reacted with (Ti,V)_2_O_3_ to generate CaTiO_3_ and (V_x_O_y_), chemically dissolved V. Concurrently, CaO reacted with silicates to produce CaAl_2_Si_2_O_8_, facilitating the sedimentation of the Fe–V alloy. Under the optimal conditions of 1873 K, a holding time of 20 min and additions of 2.5 wt% semi–coke and 12 wt% CaO, the recoveries of V, Fe and Ti reached 94.56%, 99.61% and 98.87%. In comparison, conventional processes typically achieved Fe and V recoveries of only approximately 90% and 70%. These findings support the industrial application of pre–reduced VTM.

## 1. Introduction

Vanadium (V) and titanium (Ti) are globally important strategic metals recognized by China, the United States and the European Union [1,2,3]. V and Ti are mainly hosted in vanadium–titanium magnetite (VTM) [4,5,6]. VTM is characterized by abundant reserves and wide global distribution, with proven reserves exceeding 40 billion tons [7]. It typically contains 11–58 wt% Fe, 11–21 wt% TiO_2_ and 0.2–1.9 wt% V_2_O_5_ [3,8,9,10]. Mineralogically, VTM is composed mainly of titanomagnetite, ilmenite, sulfides and gangue minerals, with gangue phases primarily consisting of sphene, chlorite, pyroxene and plagioclase. Owing to its distinctive mineralogical structure, efficient utilization of VTM remains highly challenging.

The blast furnace–converter smelting route is the principal process used for VTM treatment in China [11,12]. This route has been widely adopted because of its high maturity and large–scale production capacity. In this process, sintered VTM pellets and coke are charged into a blast furnace for high–temperature smelting at 1723–1823 K, where selective reduction and separation occur. During this stage, V is transferred to the Fe liquid phase, whereas Ti accumulates in the blast furnace slag [13,14,15]. The V–bearing hot metal is subsequently sent to the converter for blowing, where V is separated from the Fe liquid phase by oxidation and transferred to the slag. Finally, V is extracted from the slag via leaching [16]. However, the V recovery is only approximately 47%, which is unsatisfactory. Meanwhile, a large amount of blast furnace slag containing 22% TiO_2_ remains underutilized, resulting in concerns regarding resource waste. Compared with the blast furnace–converter route, the non–blast furnace smelting process for VTM has attracted increasing attention because of its potential advantages, including a shorter process flow, lower carbon emissions and higher recoveries of V and Ti. The direct reduction–electric furnace route has become a mainstream non–blast furnace process [17,18]. In this process, VTM is first pre–reduced at 1273–1373 K, during which Fe is converted to metallic iron, while Ti remains in the slag phase, to achieve the preliminary separation of Fe and Ti. The pre–reduced VTM is subjected to smelting reduction in an electric furnace to produce an Fe–V alloy and Ti–rich slag, for which the recoveries of Fe and V are 90% and 70%, respectively [19]. Approximately 80% of Ti can be extracted from Ti–rich slag through acid leaching. Nevertheless, owing to the complex mineral structure of pre–reduced VTM and the unclear transformation mechanisms of Fe, V and Ti, improvement in the recoveries of Fe, V and Ti remains difficult.

To improve the utilization efficiency of pre–reduced VTM, numerous studies have been conducted [19,20,21,22]. Zhang et al. achieved recoveries of 93.67% for Fe, 72.68% for V and 99.72% for Ti at 1473 K for 2 h, a bed thickness of 42.5 mm, a C/O molar ratio of 1.7 and the addition of 70 wt% Na_2_CO_3_ [19]. In this process, Fe oxides were reduced to metallic Fe and separated from the slag for recovery, while V was converted to water–soluble sodium vanadate. Subsequently, the slag was separated into V leachate and Ti slag via water leaching. In addition, Chen et al. reported that acid oxides in VTM could be destroyed by Na_2_CO_3_ to generate a Na–rich melt, thereby reducing slag viscosity and accelerating the reaction rate [20]. Yang et al. first pelletized a mixture of VTM with 5% coke and 3% bentonite, followed by reduction smelting at 1773 K for 30 min [21]. In that study, the total coke dosage was fixed at 15%, the basicity was adjusted by adding CaO and SiO_2_, controlled at 1.2, and an appropriate amount of CaF_2_ was introduced to regulate slag fluidity. Under the optimized conditions, efficient separation of the Fe–V alloy and Ti–rich slag was achieved, with recoveries of 99.86% for Fe, 95.64% for V and 93.54% for Ti. However, due to the high coke dosage of 15%, this process generates substantial CO and CO_2_ emissions during production. This may result in a significant carbon footprint. Although CaF_2_ is added as a flux to improve slag fluidity, its application may introduce a risk of F pollution and thus pose potential environmental hazards, making it inconsistent with the development trend of green metallurgy [22,23,24].

Furthermore, the effects of CaO and MgO additions on VTM reduction and smelting have been widely investigated. Regarding CaO addition, Cheng et al. studied the effect of calcium oxide on the crushing strength, reduction, and smelting performance of high–chromium VTM pellets [25], and Wang et al. optimized the basicity of high–Ti slag for efficient smelting of VTM metallized pellets [26]. With respect to MgO addition, Chen et al. investigated the feasibility of preparing magnesium titanate via carbothermic reduction of VTM concentrates [27] and the effects of MgO on the reduction of VTM concentrates with char [28]. Gao et al. also reported the property variations of low–grade VTM pellets with different MgO contents [29]. These studies provide valuable insights into the roles of CaO and MgO in the reduction and smelting of VTM. However, the specific migration and distribution mechanisms of Fe, V, and Ti during the electric furnace reduction smelting of pre–reduced VTM under different modifier and reductant dosages remain to be systematically clarified. Building on these studies, the present study proposes a novel smelting strategy involving VTM, CaO, MgO and semi–coke to achieve more efficient resource utilization. The objective is to clarify the transformation behavior of Fe, V and Ti and to evaluate the effects of CaO, MgO, semi–coke dosage and cooling rate, thereby supporting the industrial application of pre–reduced VTM.

Building on these studies, the present study proposed a novel smelting strategy involving VTM, CaO, MgO and semi–coke to achieve more efficient resource utilization. CaO and MgO were introduced as modifiers to release chemically dissolved V and lower the melting point of VTM. Using this strategy, Fe and V were effectively enriched in the Fe–V alloy, whereas Ti was retained in the slag. The effects of CaO, MgO and semi–coke dosage were systematically evaluated and the transformation mechanisms of Fe, V and Ti during smelting were elucidated.

## 2. Materials and Methods

**Figure 1:**
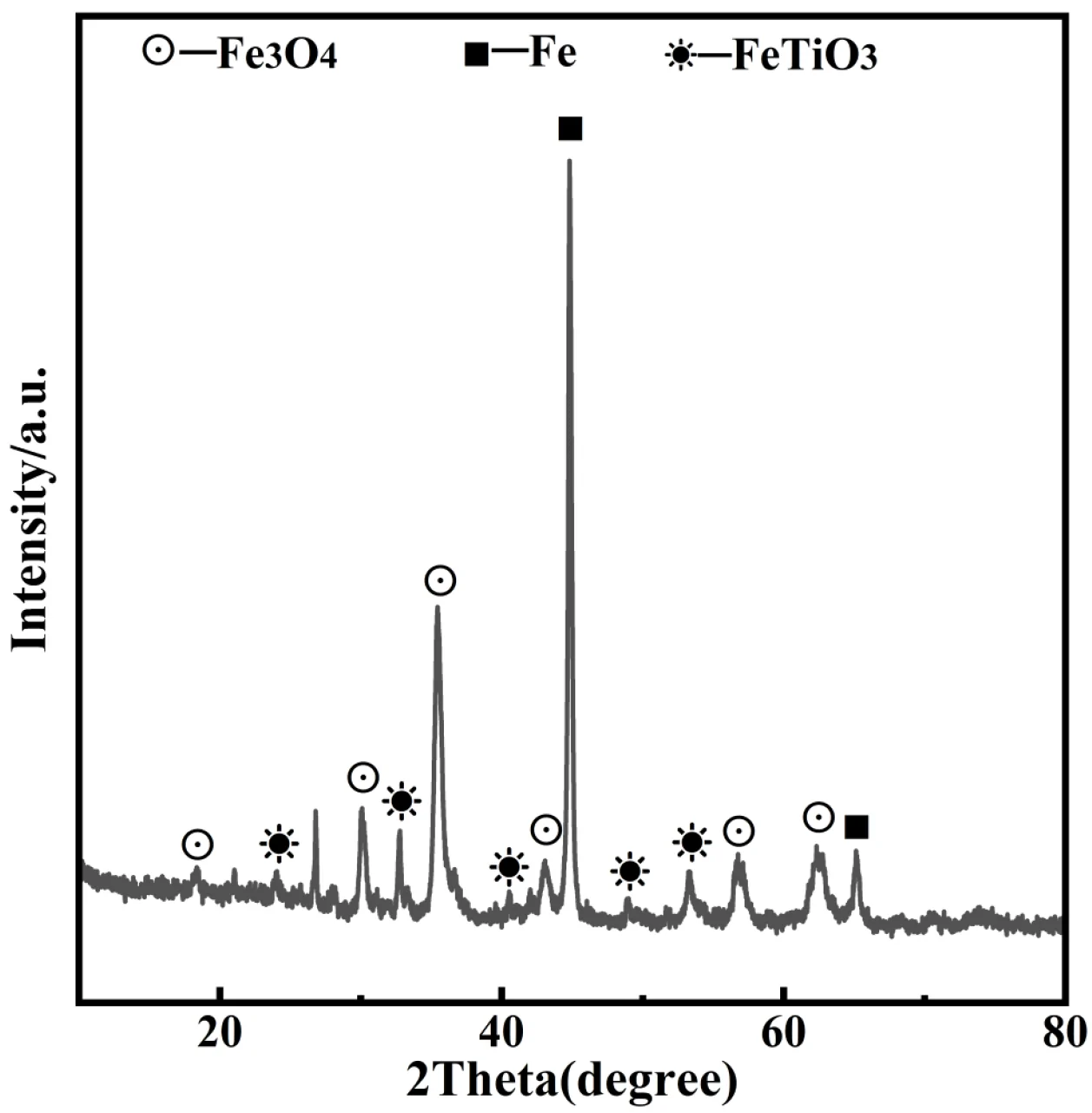
XRD pattern of the pre–reduced VTM.

**Figure 2:**
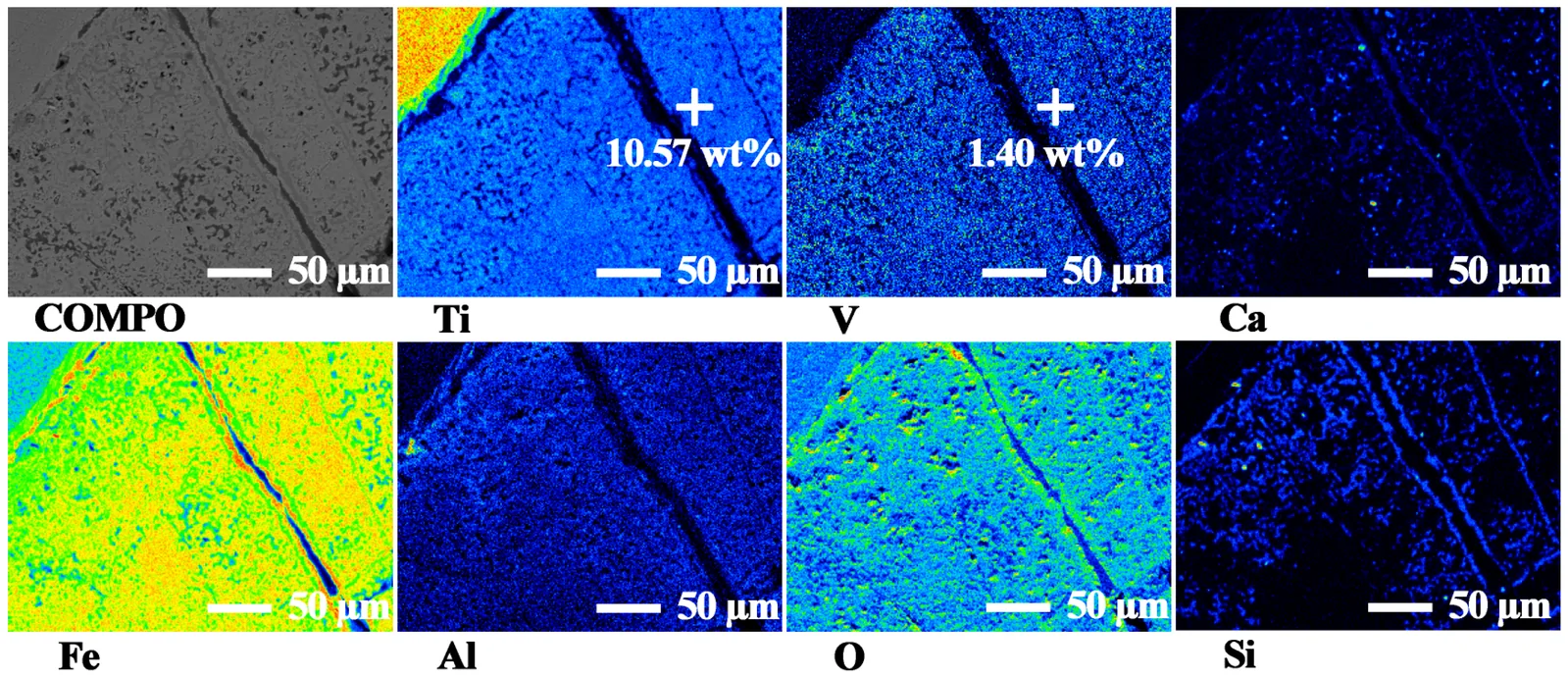
EPMA analysis of the pre–reduced VTM.

The pre–reduced VTM was obtained from a smelter in Changsha, China, composed of 57.74 wt% Fe, 1.73 wt% V_2_O_5_ and 8.81 wt% TiO_2_ (Table 1). The XRD pattern (phase analysis; Rigaku, Tokyo, Japan, TTR–Type III diffractometer) shown in Figure 1 identified Fe, FeTiO_3_ and Fe_3_O_4_ as the principal phases, whereas V was not detected because its content was too low for XRD analysis. As further revealed by the electron probe microanalysis (EPMA; JEOL, Tokyo, Japan, JXA–8230) results in Figure 2, V was mainly distributed in the FeTiO3 phase, with a measured content of 1.40 wt%. In the conventional blast furnace–converter route, Fe and V are transferred into the molten iron phase and Ti is retained in the slag and separated based on the density difference between the two phases. Semi–coke was supplied by a smelter in Yunnan, China, with 80.03 wt% fixed carbon (Table 2).

### 2.1. Methods

#### 2.1.1. Experimental Methods

**Figure 3:**
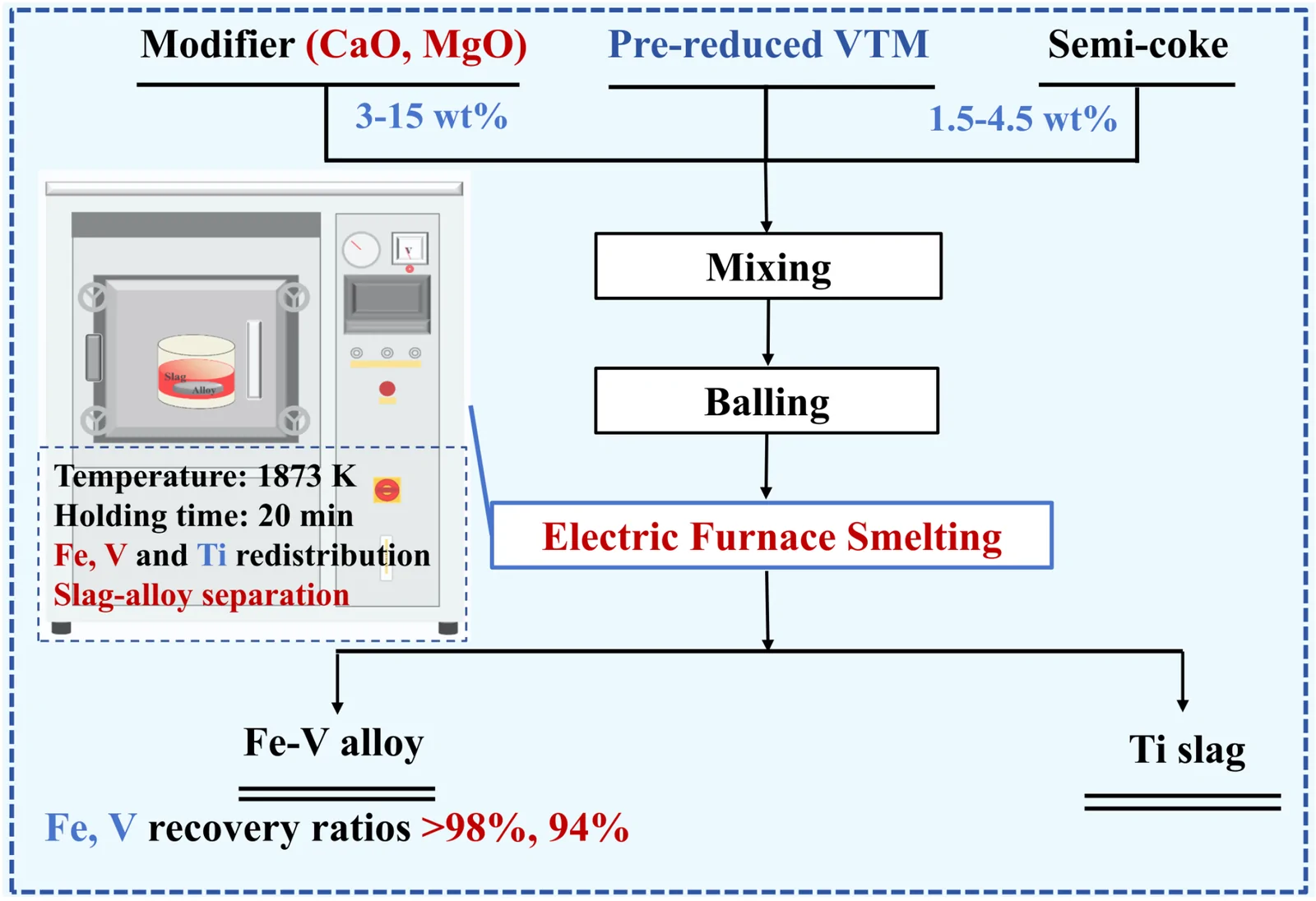
Detailed experimental procedure.

The experimental procedure is illustrated in Figure 3. Dried pre–reduced VTM powder (<75 µm), modifier (MgO or CaO) and semi–coke were blended at different mass ratios. Subsequently, 5–8 wt% water was added for pelletizing to produce pellets with a diameter of 1 cm, followed by drying at 378 K for 2 h. The additions of semi–coke and MgO/CaO were varied within the ranges of 1.5–4.5 wt% and 3–15 wt%, respectively. The prepared mixtures (approximately 100 g, 70–90 pieces) in an alumina crucible (inner radius: 30 mm; height: 100 mm) were then charged into a precision–controlled atmosphere furnace (Model: STQ–12–17, Henan Sante Furnace Technology Co., Ltd., Luoyang, China) and heated at 1873 K for 20 min under a flowing high–purity Ar atmosphere (99.90%, 200 mL/min). The furnace has internal chamber dimensions of 200 mm × 300 mm × 200 mm and a temperature control accuracy of ±1 K. The furnace is gas–tight and operates under a flowing high–purity Ar atmosphere (99.90%, 200 mL/min). A U–type thermocouple is positioned in the constant–temperature zone close to the crucible and is connected to a PID controller for temperature control. After smelting, the samples were cooled naturally to 298 K inside the furnace. The cooling occurred from approximately 1873 K to 1273 K in about 50 min, corresponding to an average cooling rate of about 12 K/min, and from 1273 K to 323 K in about 5 h.

#### 2.1.2. The Formulas and Methods

The recovery of Fe and V into the metal phase was calculated by:

Recovery_Fe,V_ (%) = [1 − (m_slag,after_ × w_Fe,V,slag,after_)/(m_feed_ × w_Fe,V,feed_)] × 100%

where m_slag,after_ is the mass of the slag after smelting, w_Fe,V,slag,after_ is the mass fraction of Fe or V in the slag after smelting, m_feed_ is the total mass of the feed (pre–reduced VTM + modifiers + reductant), and w_Fe,V,feed_ is the mass fraction of Fe or V in the feed.

The recovery of Ti into the slag phase was calculated by:

Recovery_Ti_ (%) = (m_slag,after_ × w_Ti,slag,after_)/(m_feed_ × w_Ti,feed_)] × 100%

where w_Ti,slag,after_ is the mass fraction of Ti in the alloy after smelting and w_Ti,feed_ is the mass fraction of Ti in the feed.

#### 2.1.3. Characterization

To ensure analytical reliability, chemical compositions were determined using the standard chemical methods GB/T14506.32–2019 [30] and GB/T14353.1–2010 [31], along with inductively coupled plasma optical emission spectrometry (ICP–OES, YB/T 4177.2–2025) [32]. Each sample was measured in triplicate, and the reported values represent the average of the three measurements. Phase identification was achieved by X–ray diffraction (XRD) using a RIGAKU TTRIII–18KW diffractometer (Rigaku, Tokyo, Japan) operated at 40 kV and 40 mA with Cu–Kα radiation. The scans were collected over 10–90° at a scanning rate of 10°/min and a step size of 0.02°. Moreover, EPMA coupled with energy–dispersive X–ray spectroscopy (EDS, JEOL, Tokyo, Japan, JXA–8230) was employed to characterize the phase evolution and elemental distribution in the samples under operating conditions of 10–25 kV and 2–50 nA.

## 3. Thermodynamic Analysis

### 3.1. Carbothermal Reduction Smelting of Pre–Reduced VTM

**Figure 4:**
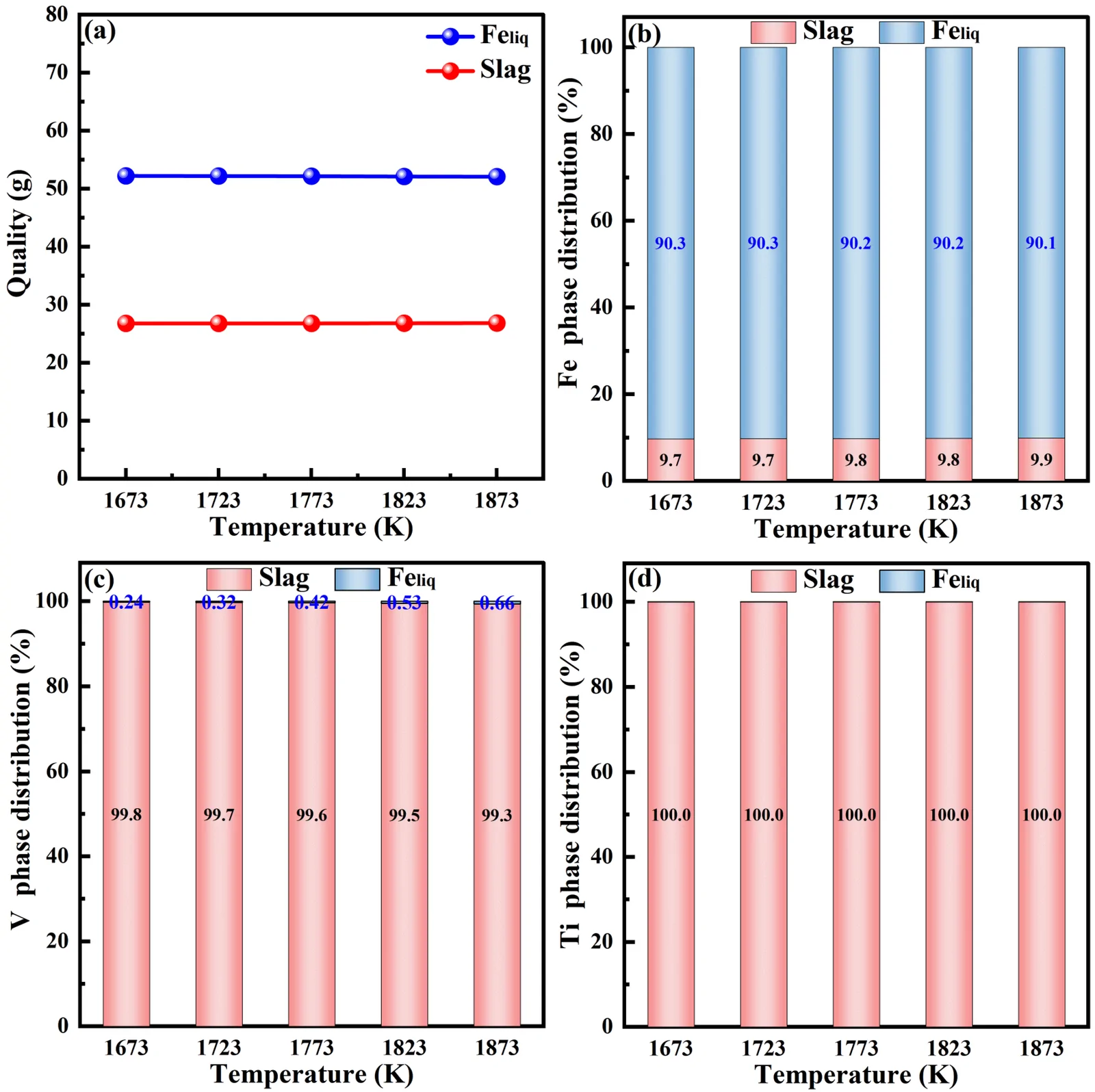
Calculated effect of temperature on the multicomponent complex thermodynamics during smelting with 2 wt% C addition: (**a**) phase distribution; (**b**) distribution behavior of Fe phases; (**c**) distribution behavior of V phases; (**d**) distribution behavior of Ti phases.

Multiphase equilibrium calculations based on the process mineralogy of pre–reduced VTM (FactSage 8.1 Equilib module; Table 3 and Table 4, and Figure 4) indicated that at a C addition of 2 wt%, the equilibrium products were dominated by the Fe_liq_ phase and slag. Temperature appears to slightly affect the distribution of V and C, and strongly affect viscosity. More than 90% of Fe was distributed in the Fe_liq_ phase, with an Fe grade exceeding 99 wt% (Table 5). In comparison, the V recovery improved only slightly, increasing from 0.24 to 0.66% as the temperature increased, while the slag still contained more than 4 wt% V (Table 6), indicating poor V recoverability. On the other hand, Ti was completely recovered over 1673–1873 K, and its concentration in the slag increased to 19.72 wt% Ti (Table 6). Additionally, the slag viscosity decreased markedly from 0.100 to 0.064 Pa·s with increasing temperature (FactSage Viscosity module; Table 4, Table 5, Table 6 and Table 7).

**Figure 5:**
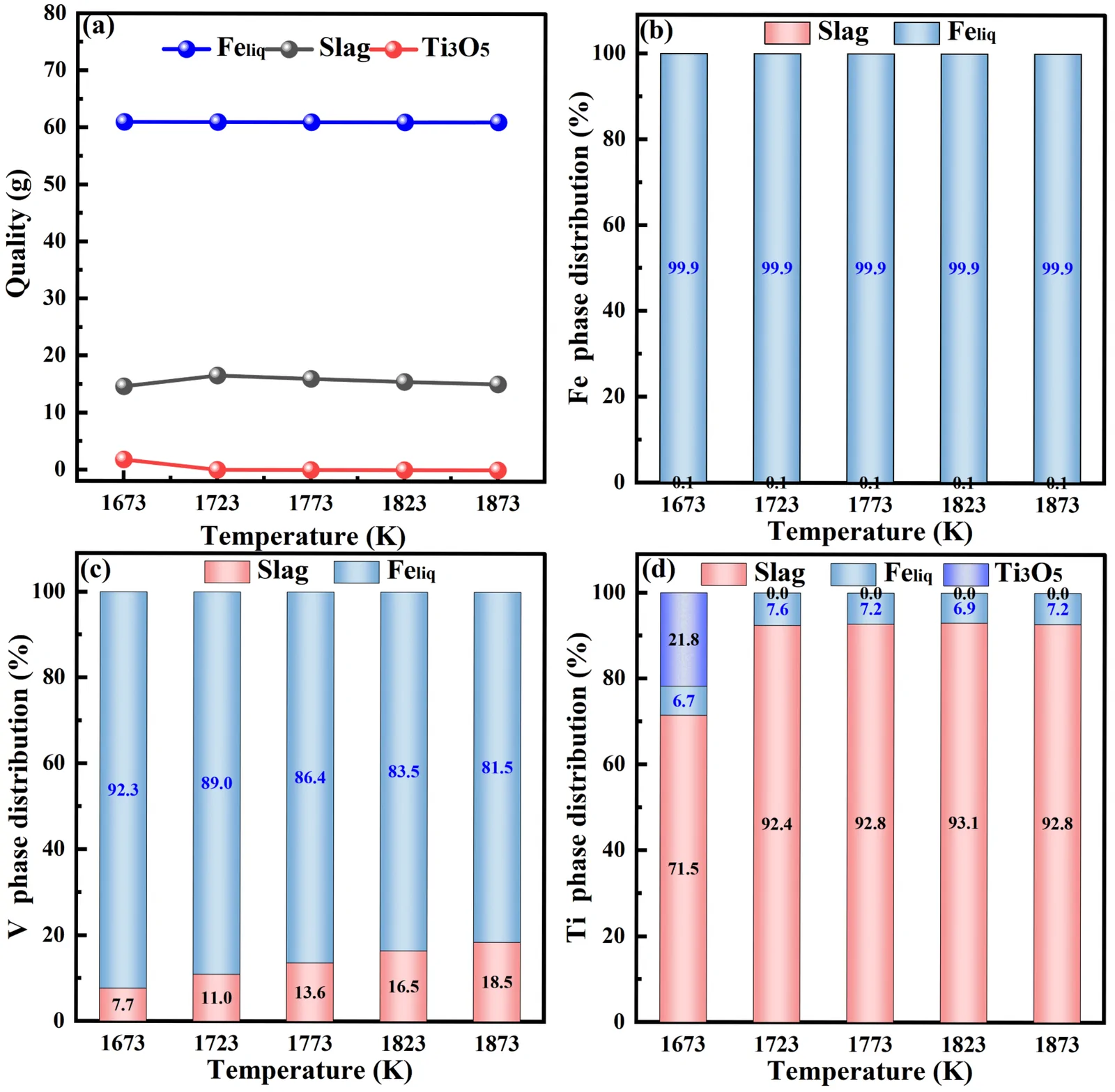
Calculated effect of temperature on the multicomponent complex thermodynamics during smelting with 6 wt% C addition: (**a**) phase distribution; (**b**) distribution behavior of Fe phases; (**c**) distribution behavior of V phases; (**d**) distribution behavior of Ti phases.

As shown in Figure 5, when 6 wt% C was added at 1673 K, 81% of V was transferred to the Fe_liq_ phase, leaving only 0.30% of V in the slag. When the temperature was further increased to 1873 K, the fraction of V distributed in the Fe_liq_ phase decreased to 89.3%, whereas the V content in the slag increased to 0.75%. Throughout the investigated temperature range, more than 89% of V entered the Fe_liq_ phase, along with 18% of Ti. These results indicate that increasing the C dosage enabled the simultaneous and efficient recovery of Fe, V and Ti, exceeding 99%, 89% and 82%, respectively. The Fe_liq_ phase contained 94 wt% Fe and 1.2 wt% V (Table 8), whereas the impurity levels of Si and C remained low. Meanwhile, the Ti content in the slag phase increased to more than 32% (Table 9). However, the reduction of (FeO) in the slag caused a pronounced increase in the slag viscosity with increasing temperature (Table 7, Table 8, Table 9 and Table 10), which severely hindered mass transfer and slag–metal separation. The slag system must be optimized.

**Figure 6:**
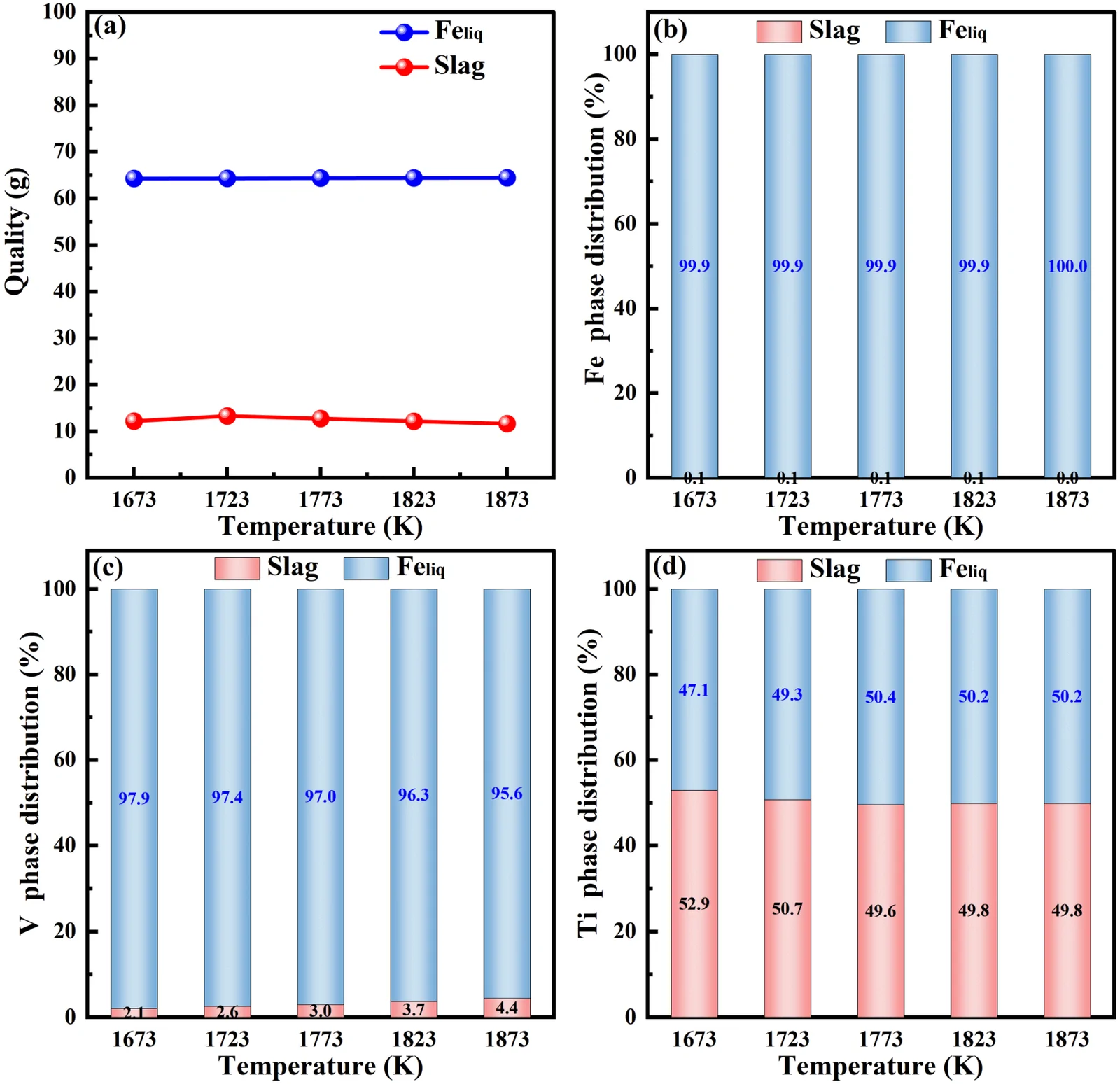
Calculated effect of temperature on the multicomponent complex thermodynamics during smelting with 8 wt% C addition: (**a**) phase distribution; (**b**) distribution behavior of Fe phases; (**c**) distribution behavior of V phases; (**d**) distribution behavior of Ti phases.

When the C addition was increased to 8 wt%, a large amount of Ti was added into molten iron (Figure 6), indicating that over–reduction occurred and that the slag–metal separation during smelting consequently deteriorated. Considering the overall process performance, the optimal C addition was determined to be 6 wt%. These results suggest that the reduction sequence of valuable metals in pre–reduced V titanomagnetite follows the order: Fe_3_O_4_ > (FeO)_slag_ > (V_2_O_5_)_slag_ > (Ti_2_O_3_)_slag_.

### 3.2. Slag Engineering for Carbothermal Smelting of Pre–Reduced VTM

To improve the slag viscosity and metal–slag separation while reducing the smelting temperature, CaO and MgO, which are the most widely used industrial additives, were introduced to examine their effects on slag physicochemical properties and valuable–metal recovery.

#### 3.2.1. Thermodynamic Effect of CaO

**Figure 7:**
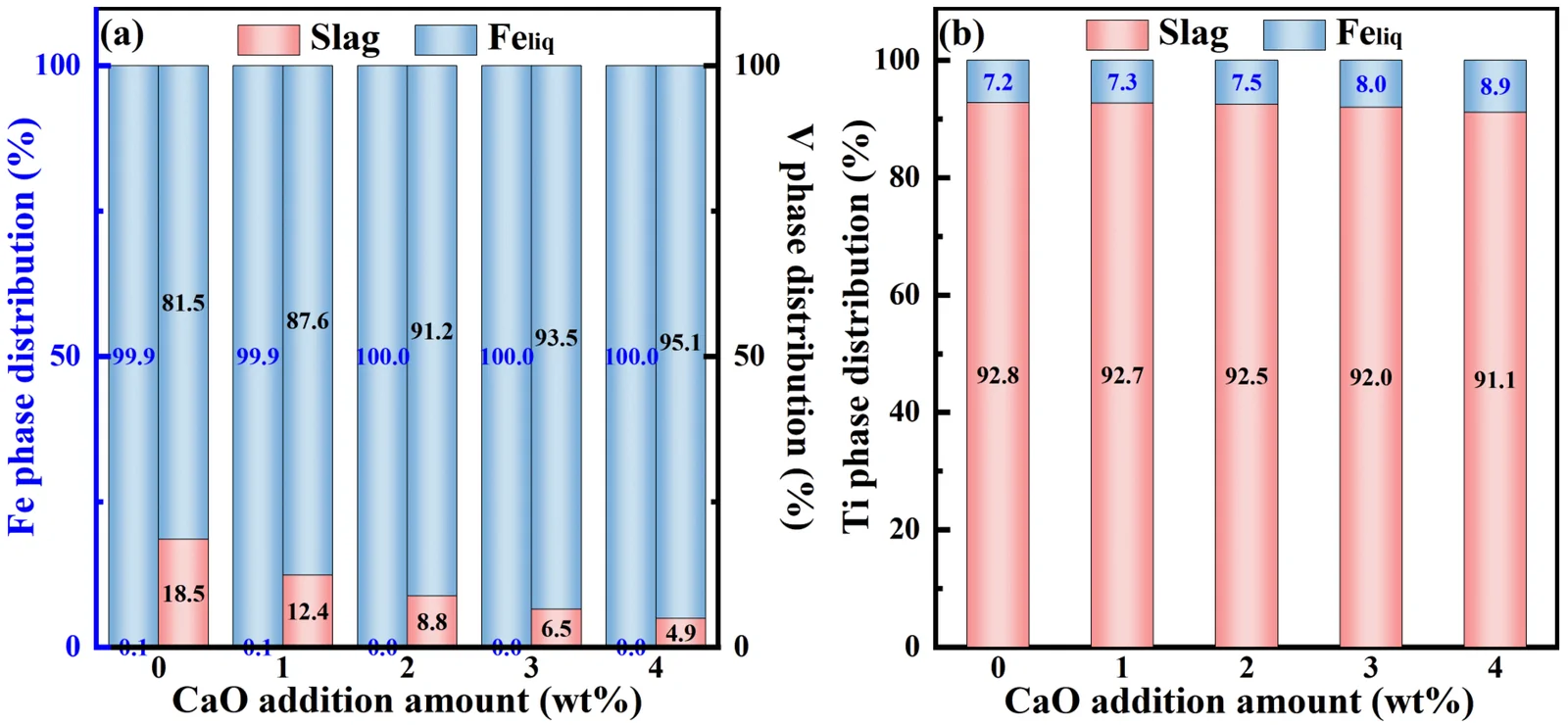
Calculated effect of temperature on the multicomponent complex thermodynamics during smelting: (**a**) distribution behaviors of Fe and V phases; (**b**) distribution behavior of Ti phases.

Figure 7 and Table 11, Table 12 and Table 13 present the effects of CaO addition (0–4 wt%) at 1873 K on the distributions of Fe, V and Ti and on the elemental compositions of the Fe_liq_ and slag phases. The Fe distribution was barely influenced by CaO addition, and more than 99.80% of Fe remained in the Fe_liq_ phase throughout the tested range. In contrast, the transfer of V to the Fe_liq_ phase was continuously enhanced, with its distribution ratio increasing from 81.5% to 95.1% as the CaO addition increased from 0 to 4 wt%. In parallel, the V content in the Fe_liq_ phase increased from 1.296 wt% to 1.508 wt% (Table 12), indicating that CaO promoted the migration of V into the Fe_liq_ phase. Once the CaO dosage exceeded 3 wt%, the V recovery rose above 93.5% (Figure 7). In comparison, the Ti recovery exhibited minimal variation, remaining at approximately 91.1%, whereas the TiO_2_ content in the slag remained above 24% (Table 12). Meanwhile, the slag viscosity decreased sharply from 0.100 to 0.048 Pa·s (Table 13), which facilitated the separation of the Fe liquid from the slag. Overall, the CaO addition promoted valuable metal recovery during smelting, and the optimal dosage was determined to be 3 wt% based on economic factors.

#### 3.2.2. Thermodynamic Effect of MgO

**Figure 8:**
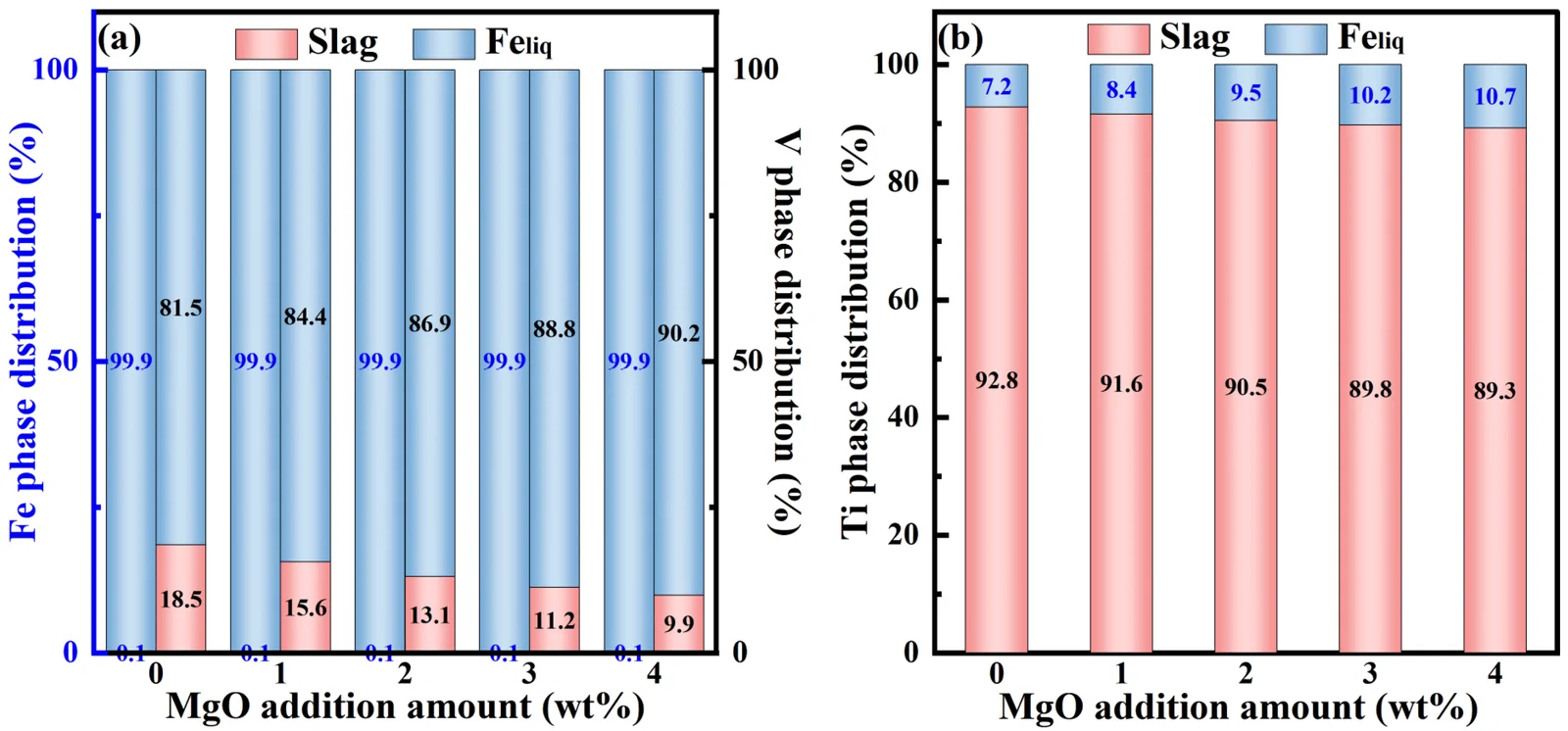
Calculated effect of temperature on the multicomponent complex thermodynamics during smelting, with 6 wt% C addition: (**a**) distribution behaviors of Fe and V phases; (**b**) distribution behavior of Ti phases.

Figure 8 and Table 14, Table 15 and Table 16 present the effects of MgO addition (0–4 wt%) at 1873 K on the distributions of Fe, V and Ti, the elemental compositions of the Fe_liq_ and slag phases, and the crystalline phases of the slag at 1873 K. Similar to CaO, the increasing MgO addition improved V recovery. It increased from 81.5% to 90.2%, indicating that MgO also facilitated V transfer. However, the Ti partitioning to the Fe_liq_ phase also increased, rising from 7.2% to 10.7%, which was unfavorable for Ti recovery. Moreover, the slag viscosity decreased from 0.100 to 0.060 Pa·s as the MgO content increased (Table 16). These results indicate that MgO improved V recovery but weakened Ti recovery, and its effect on reducing slag viscosity was less pronounced than that of CaO. Therefore, when both recoveries and economic factors are considered, the MgO addition at 1673 K should be limited to below 3 wt%.

## 4. Results and Discussion

### 4.1. Effect of MgO

**Figure 9:**
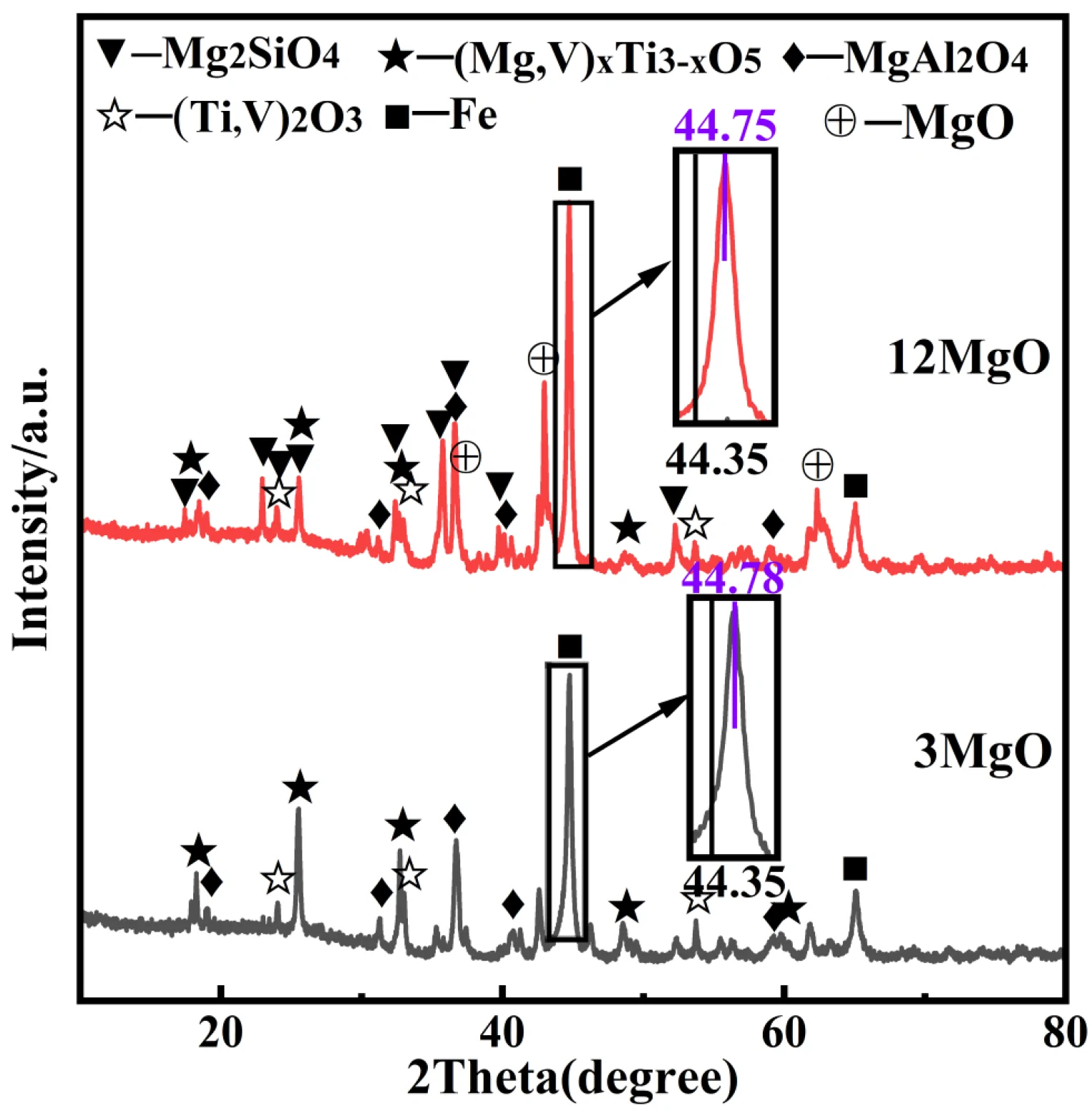
XRD pattern of slag with MgO additions.

**Figure 10:**
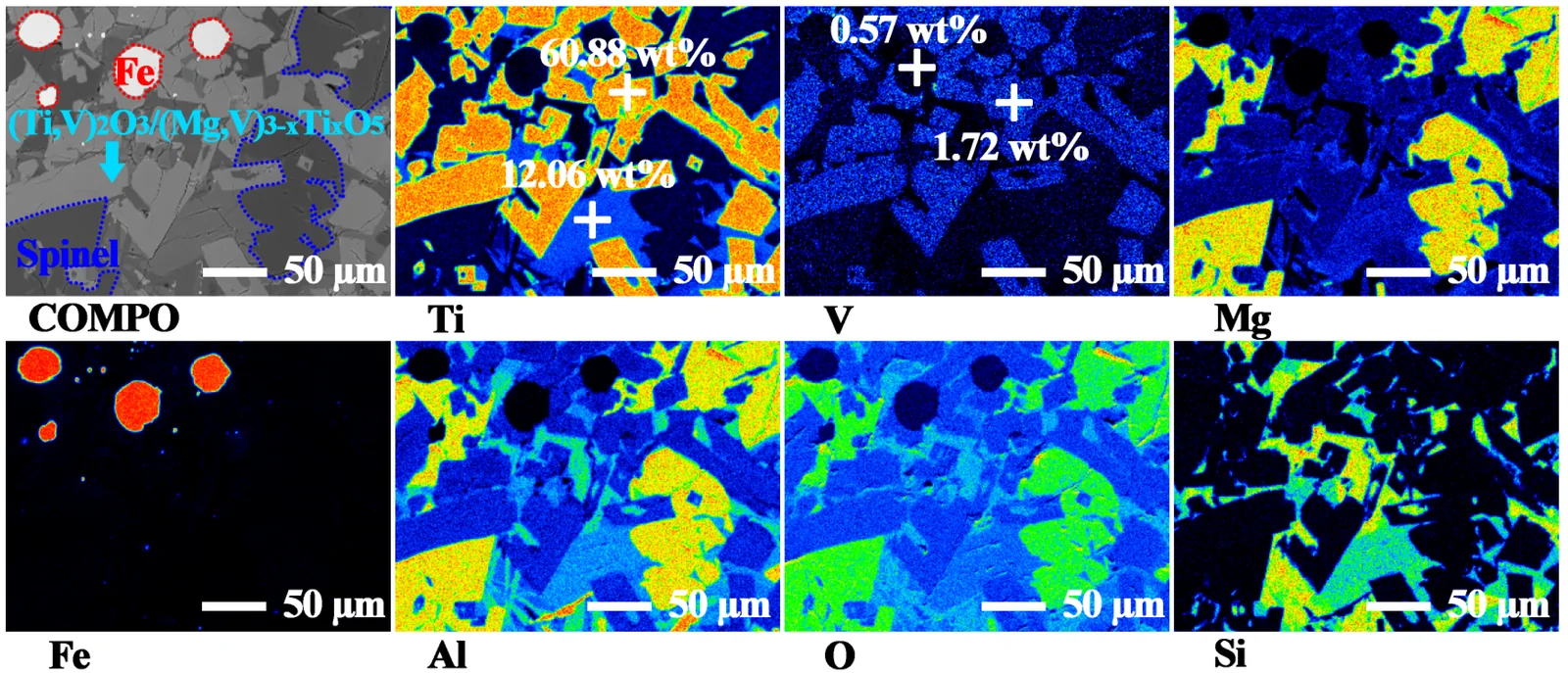
EPMA analysis of slag with 3 wt% MgO addition.

A mixture of 2.5% semi–coke, 3–12 wt% MgO and pre–reduced VTM powder was thoroughly blended, pelletized in a crucible and held at 1873 K for 20 min to investigate the effect of MgO addition on the recovery of V, Fe and Ti from pre–reduced VTM. Under the condition of 3 wt% MgO addition, slag–alloy separation was not observed (Table 17), which differed markedly from the thermodynamic prediction in Section 3.2.2. XRD results (Figure 9) showed that the main phases in the slag were (Mg,V)_x_Ti_3–x_O_5_ (melting point: 2453 K), spinel (MgAl_2_O_4_, melting point: 2408 K), (Ti,V)_2_O_3_ (melting point: 2403 K) and Fe. Comparing Figure 1 and Figure 9, the disappearance of FeTiO_3_ and the appearance of (Ti,V)_2_O_3_ and (Mg,V)_x_Ti_3–x_O_5_ suggest that FeTiO_3_ was first reduced by C to Fe, V_x_Ti_3–x_O_5_ and (Ti,V)_2_O_3_. In contrast, the added MgO entered these solid solutions and replaced the lattice V through a reaction with both phases. This process promoted the release of V and its conversion into chemically dissolved species, thereby facilitating subsequent reduction by C and transfer into the Fe–V alloy. However, because MgAl_2_O_4_, (Ti,V)_2_O_3_ and (Mg,V)_x_Ti_3–x_O_5_ possess high melting points, their melting at 1873 K is difficult, which hinders the settling of the alloy, thereby suppressing the recovery of Fe and V. As shown in Figure 10, Fe existed in the form of a Fe–V alloy, whereas the slag phase contained almost no Fe. In addition, V was primarily concentrated in the (Mg,V)_x_Ti_3–x_O_5_ and (Ti,V)_2_O_3_ phases, with a content of 1.72 wt%, whereas the V content in the Fe–V alloy was only 0.57 wt%, corresponding to a predicted V recovery of only 30% under the assumption of slag–alloy separation.

**Figure 11:**
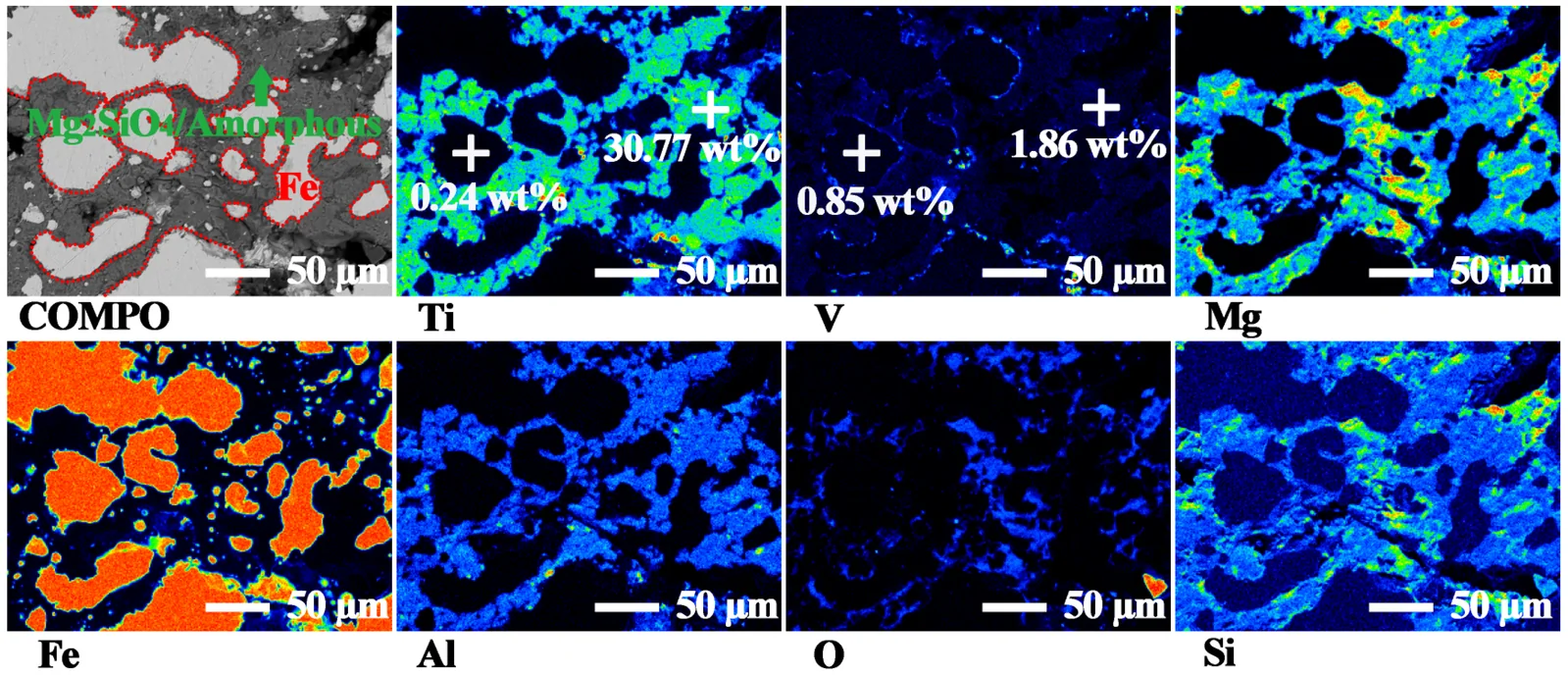
EPMA analysis of slag with 12 wt% MgO addition.

When the MgO addition was increased to 12 wt%, the slag–alloy separation became even worse. XRD analysis demonstrated that the intensity of Fe diffraction peaks in the slag increased, and the Fe diffraction peak shifted from 44.35° to 44.75°, which may be attributed to the entry of V with a smaller atomic radius into the Fe lattice. In addition, numerous Mg_2_SiO_4_ diffraction peaks appeared (Figure 9, 2163 K). The comparison of the electron microprobe analyses in Figure 10 and Figure 11 showed that the size of the Fe–V alloy particles in the slag increased from 34 μm to more than 200 μm. This increase was attributed to the higher content of the amorphous phase, which reduced the slag viscosity and facilitated the aggregation and growth of Fe–V alloy particles. Meanwhile, the V content in the Fe–V alloy increased from 0.57 wt% to 0.85 wt%. This result further indicates that MgO addition disrupted the crystal structure of (Ti,V)_2_O_3_ and released chemically dissolved V, which was subsequently reduced by C into the Fe–V alloy. However, excessive MgO also promoted the formation of Mg_2_SiO_4_ in the slag, thereby increasing slag viscosity and preventing the Fe–V alloy from settling. Therefore, the application of MgO as a modifier was not favorable for slag–alloy separation or V recovery.

### 4.2. Effect of CaO

**Figure 12:**
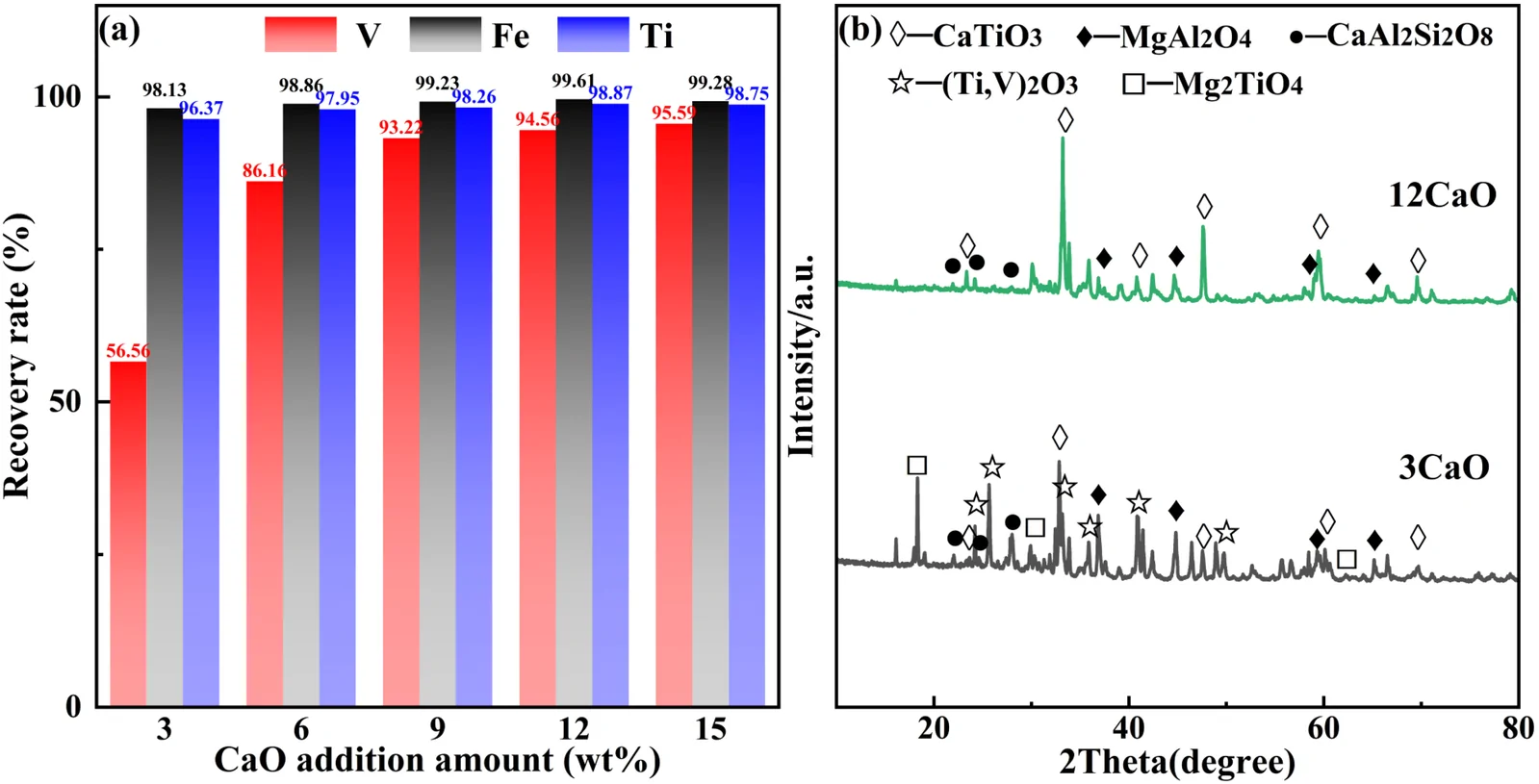
Effect of CaO addition on (**a**) the recovery of V, Fe and Ti; (**b**) XRD pattern of slag.

**Figure 13:**
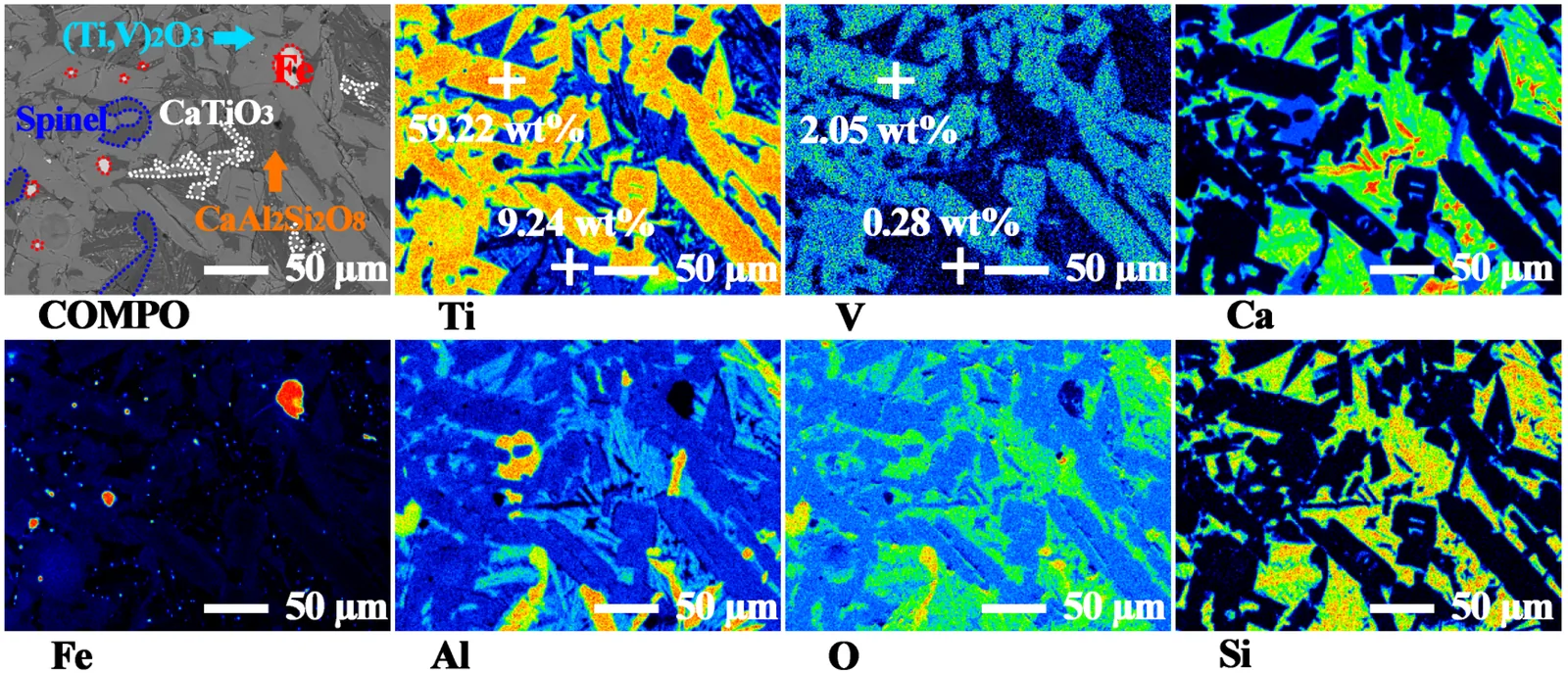
EPMA analysis of slag with 3 wt% CaO addition.

A mixture of 2.5% semi–coke, 3–15 wt% CaO and pre–reduced VTM powder was thoroughly blended and granulated in a crucible. The samples were then held at 1873 K for 20 min to simulate industrial production conditions, and the effect of CaO addition on the recovery of V, Fe and Ti from pre–reduced VTM was investigated. Unlike the case with 3 wt% MgO addition, outstanding slag–alloy separation was achieved with 3 wt% CaO addition. As shown in Table 18, the V content in the slag with 3 wt% CaO was 1.35 wt%, corresponding to a V recovery of 56.56%, while the recoveries of Fe and Ti reached 98.13% and 96.37%, respectively. These results indicate that CaO as a modifier is more favorable for V recovery and improves the slag–alloy separation condition. A comparison of Figure 9 and Figure 12b indicates that the Fe diffraction peaks disappeared from the slag, whereas CaAl_2_Si_2_O_8_ (calcium feldspar, melting point: 1826 K) appeared. This suggests that the liquid CaAl_2_Si_2_O_8_ formed at 1873 K improved the slag mobility and rendered the slag–alloy separation possible. As shown in Figure 13, only a few small metallic Fe particles (<10 μm) remained in the slag, and the V contents in (Ti,V)_2_O_3_ and CaAl_2_Si_2_O_8_ were 2.05 wt% and 0.28 wt%, respectively. In accordance with the thermodynamic stepwise reduction analysis, FeTiO_3_ in the pre–reduced VTM was first reduced by C to Fe and V_x_Ti_3–x_O_5_, after which V_x_Ti_3–x_O_5_ was reduced by C to form (Ti,V)_2_O_3_. The added CaO then reacted with (Ti,V)_2_O_3_ to form CaTiO_3_ and release (V_x_O_y_)_chemically dissolved V_, which was subsequently reduced again by C and transferred to the Fe–V alloy (Figure S1).

**Figure 14:**
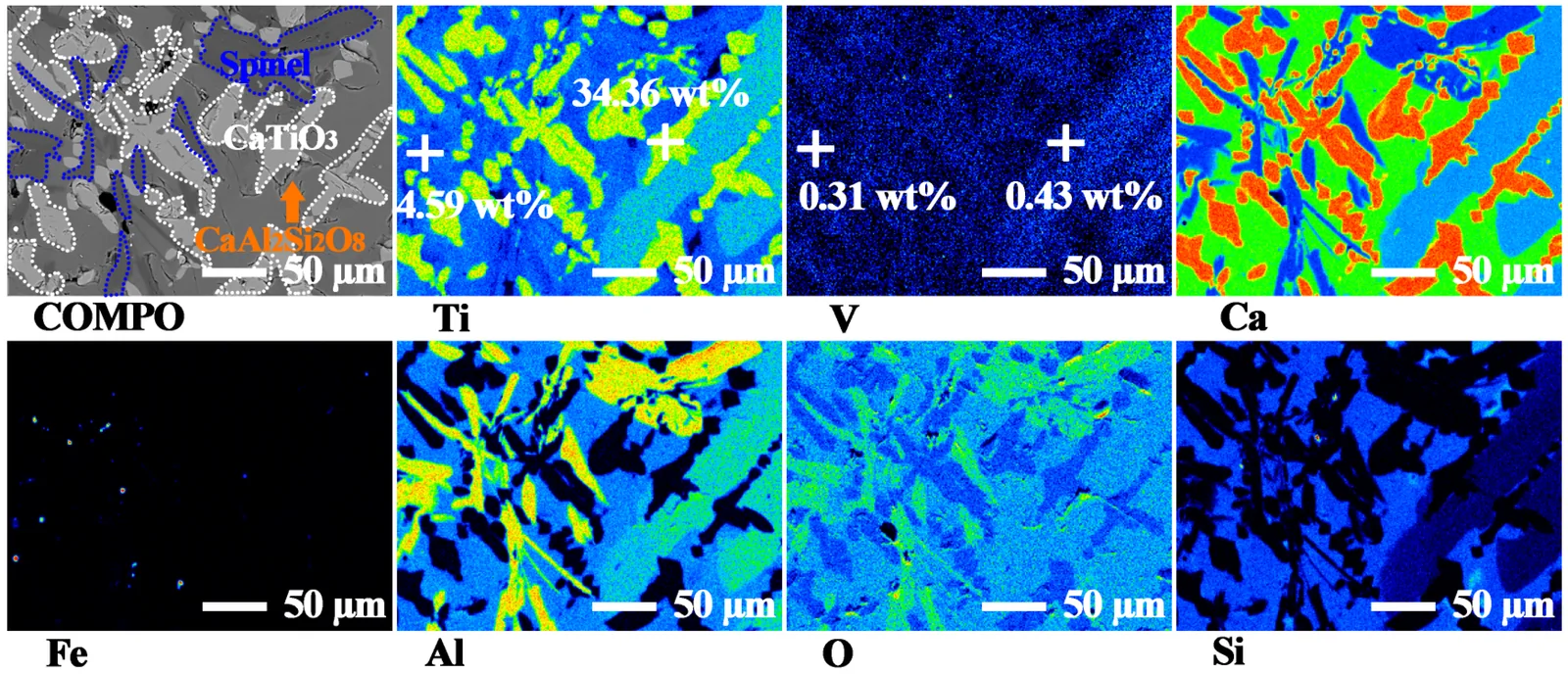
EPMA analysis of slag with 12 wt% CaO addition.

As the CaO addition gradually increased to 12 wt%, the V content in the slag decreased from 1.35 wt% to 0.20 wt% (Table 18). Meanwhile, the V recovery increased from 56.56% to 94.56% (Figure 12a). The recoveries of Fe and Ti remained above 98% and 96%, respectively. Similar to the role of MgO, this behavior indicates that CaO disrupts the crystal structure of (Ti,V)_2_O_3_ and promotes the release of chemically dissolved V for reaction with C. The XRD results in Figure 12b revealed that as the CaO addition increased to 12 wt%, the diffraction peaks of (Ti,V)_2_O_3_ disappeared. In contrast, the diffraction peaks of CaTiO_3_ gradually appeared and intensified. At this stage, only CaTiO_3_, spinel and calcium feldspar were detected. Figure 14 illustrates the disappearance of (Ti,V)_2_O_3_ and the formation of CaTiO_3_. At this point, the bulk (Ti,V)_2_O_3_ phase (>150 μm) disappeared and was replaced by CaTiO_3_, and the detected V signal was identified only in spinel and CaTiO_3_ at 0.31 wt% and 0.43 wt%, respectively. This marked decrease in V content compared with the 3 wt% CaO condition was consistent with the reduction in slag V content from 1.35 wt% to 0.19 wt% with increasing CaO content (Table 18). These results confirmed that the CaO addition can promote V reduction and improve V recovery. Considering the recoveries of V, Fe and Ti with economic benefits, the optimal CaO addition was determined to be 12 wt%. Considering the MgO–based furnace lining, the simultaneous effect of CaO and MgO was further evaluated. With the addition of 0.5 wt% MgO on the basis of 12 wt% CaO, the slag contained 0.20 wt% V and 0.23 wt% Fe, and the Ti content in the alloy was 0.16 wt%. These results are almost identical to those obtained without MgO addition, indicating that a minor amount of MgO from the lining has a negligible effect on the separation of Fe, V and Ti.

### 4.3. Migration and Transformation of Fe, V, and Ti During the Smelting Process

**Figure 15:**
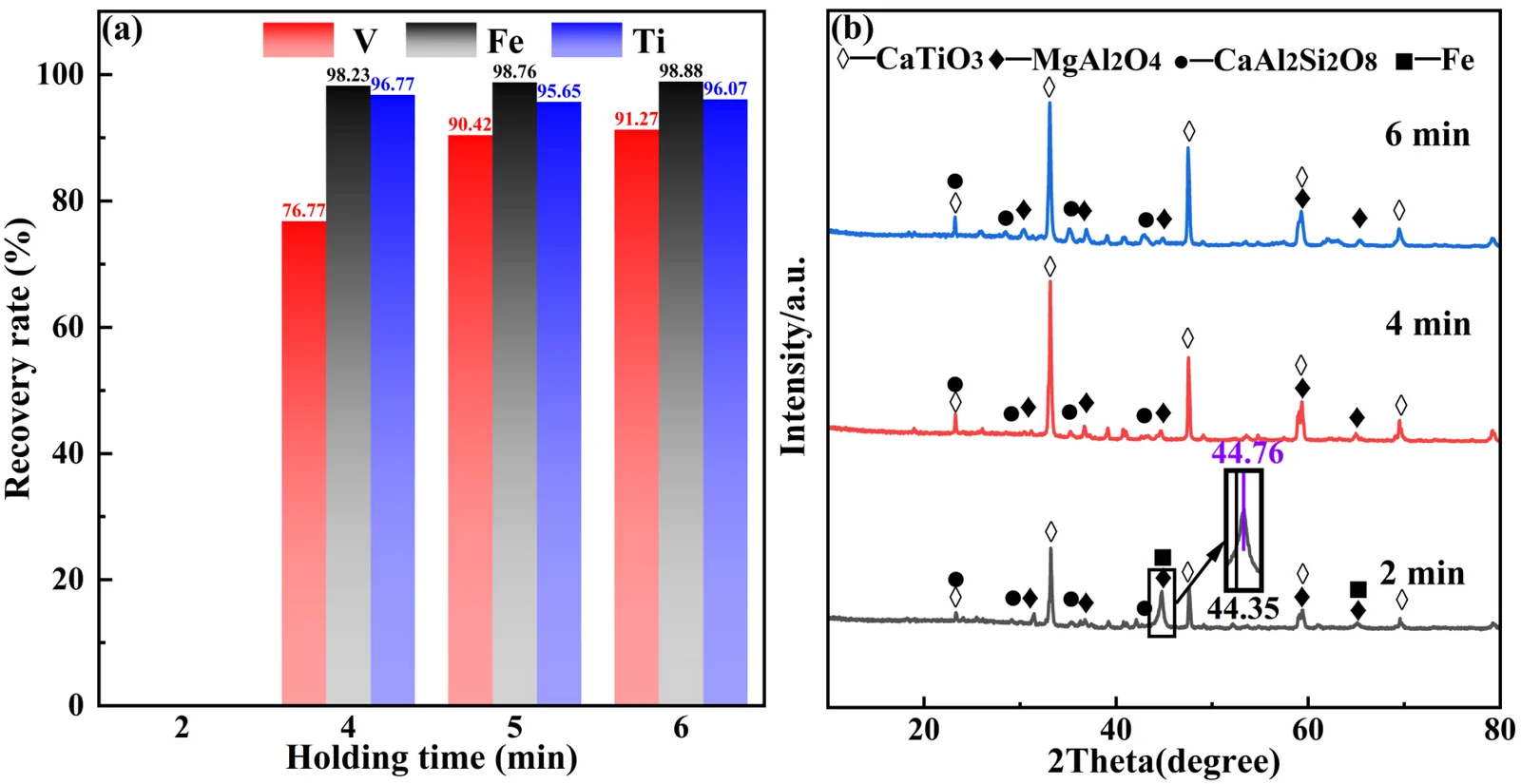
Effect of holding time on (**a**) the recovery of V, Fe and Ti; (**b**) XRD pattern of slag.

**Figure 16:**
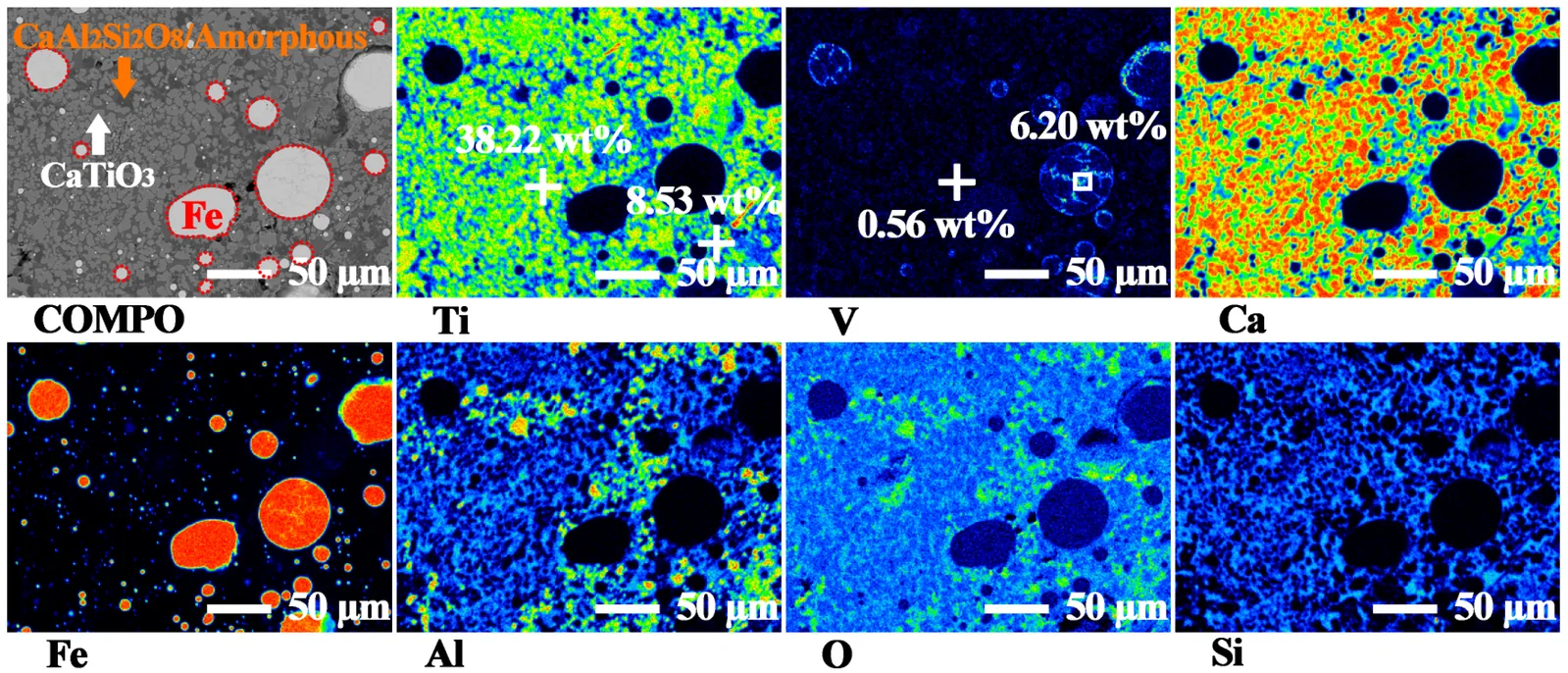
EPMA analysis of slag for holding 2 min.

**Figure 17:**
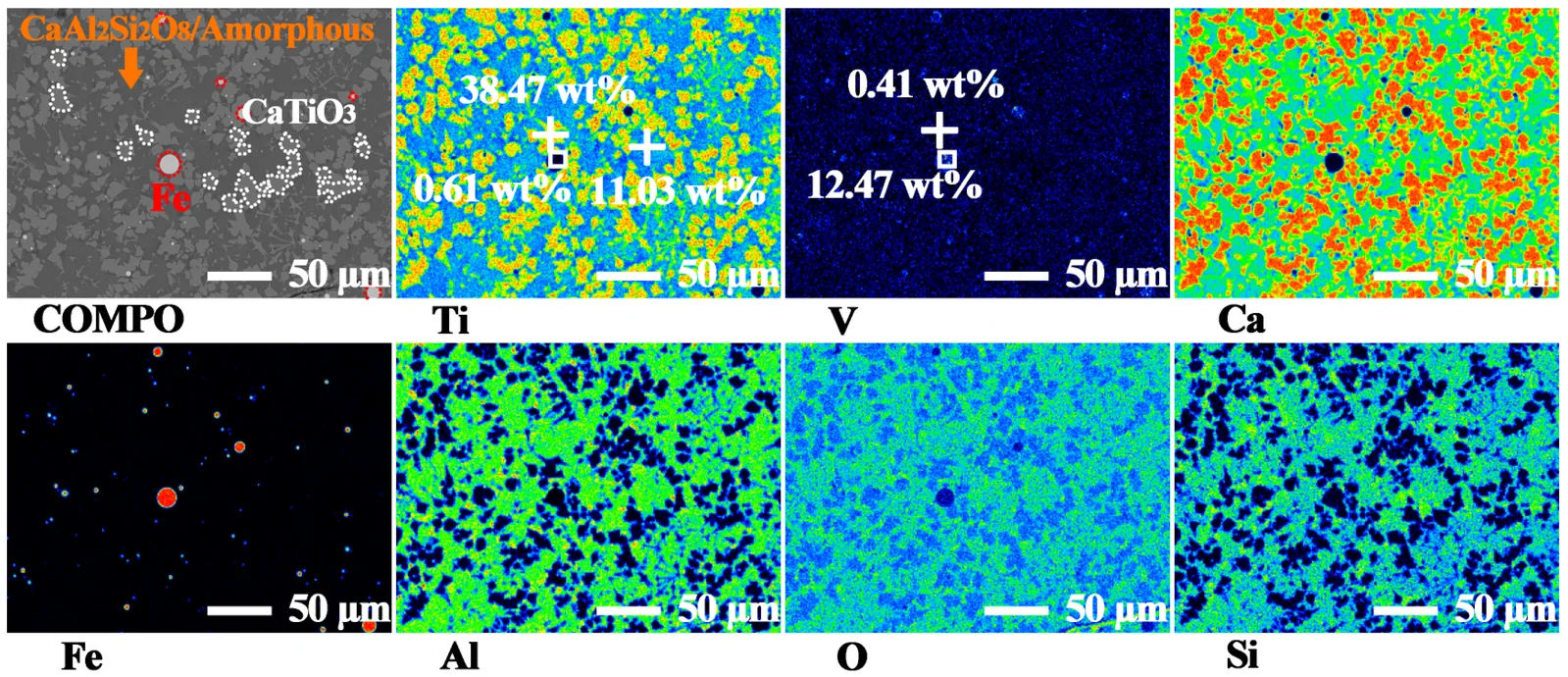
EPMA analysis of slag after holding for 4 min.

**Figure 18:**
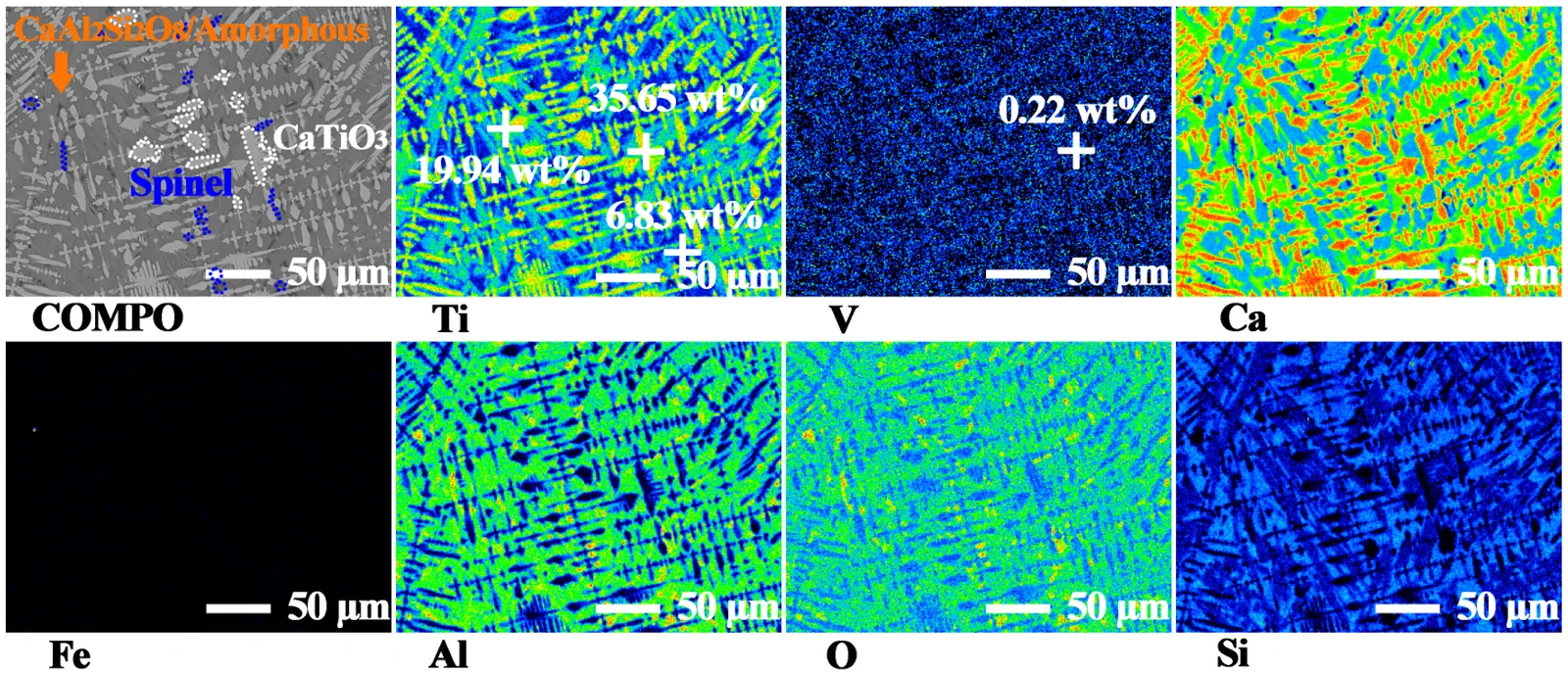
EPMA analysis of slag after holding for 6 min.

CaO and pre–reduced VTM powder was thoroughly blended, pelletized in a crucible and then rapidly water–quenched after holding at 1873 K for 2, 4, 5 and 6 min, respectively. The detailed results are presented in Table 19 and Figure 15, Figure 16, Figure 17 and Figure 18. Owing to the brief holding time of 2 min, the slag entered the smelting zone but did not reach the target temperature of 1873 K, causing incomplete melting and no effective recovery of V, Fe or Ti (Table 19). The XRD pattern in Figure 15b showed that the slag at this stage consisted of CaTiO_3_, CaAl_2_Si_2_O_8_ and Fe. Because V doping shifted the Fe diffraction peak from the standard position of 44.35° to 44.76°, V remained undetectable by XRD at low concentrations. EPMA of the slag (Figure 16) indicated that the main phases were Fe, CaTiO_3_ and CaAl_2_Si_2_O_8_, with the V content in metallic Fe reaching 6.20 wt%. Combined with the reaction sequence of FeTiO_3_, these observations indicated that the stepwise reduction of FeTiO_3_ generated (Ti,V)_2_O_3_, which then reacted with the added CaO (Equation (1)) to form CaTiO_3_ while simultaneously releasing chemically dissolved V. This dissolved V remained in the slag and contacted carbon, where it was reduced to metallic Fe (Equation (2)). This explains why the V content in CaTiO_3_ was only 0.56 wt%, whereas 6.20 wt% V was identified in Fe.

(Ti,V)_2_O_3_ + CaO → CaTiO_3_ + (V_x_O_y_)_chemically dissolved V_ (1)

(V_x_O_y_)_chemically dissolved V_ + C = [V]_Fe–V alloy_ + CO (2)

When the holding time was increased to 4 min, the slag began to melt and the slag–alloy separation started. As shown in Table 19, the V content in the slag decreased to 0.55 wt%, and the corresponding V recovery increased to 76.77%. The XRD results showed that the Fe diffraction peaks disappeared, whereas the diffraction peaks of CaTiO_3_, CaAl_2_Si_2_O_8_ and spinel remained. This indicates that the slag contained almost no metallic Fe and confirms that Fe–V alloy sedimentation had been essentially completed. However, because a considerable amount of V remained, further recovery at this stage was mainly controlled by kinetics. Only a very small amount of metallic Fe (<12 μm) was present, and the main phases were CaTiO_3_ and CaAl_2_Si_2_O_8_, with V contents of only 12.47 wt% each. Combining Figure 15 and Figure 17, the intensities of the diffraction peaks of CaAl_2_Si_2_O_8_ were low, suggesting poor crystallinity. The comparison of Figure 16 and Figure 17 indicates that the V content in Fe increased markedly from 6.20 wt% to 12.47 wt%, whereas the V content in CaTiO_3_ decreased from 0.56 wt% to 0.41 wt%, further confirming the occurrence of Equation (1). When the holding time was extended to 6 min, the V content in the slag was further reduced to 0.23 wt% (Table 19), and the corresponding V recovery increased to 91.27%. The XRD pattern (Figure 15) showed no significant change in the diffraction peaks, and the main slag phases remained CaTiO_3_ and CaAl_2_Si_2_O_8_. Compared with Figure 17 and Figure 18, the morphology of CaTiO_3_ changed from granular to flocculent. This was because the slag had completely melted with prolonged holding for 6 min, whereas CaTiO_3_ precipitated during water quenching owing to its higher melting point. At this stage, the Fe–V alloy in the slag had completely disappeared, and the V content in CaTiO_3_ decreased from 0.41 wt% to 0.22 wt%. When the holding time was shorter than 6 min, CaTiO_3_ in the slag was not fully melted because of kinetic limitations. Consequently, V in the CaTiO_3_ lattice was difficult to reduce. The slag was completely melted only after holding for 6 min, allowing the chemically dissolved V and carbon to be fully contacted and the reduction reaction to proceed. It eliminated the kinetic barrier associated with low–temperature diffusion of chemically dissolved V to the slag–alloy interface, where it reacted with carbon, entered the Fe–V alloy and improved V recovery.

### 4.4. Effect of C

**Figure 19:**
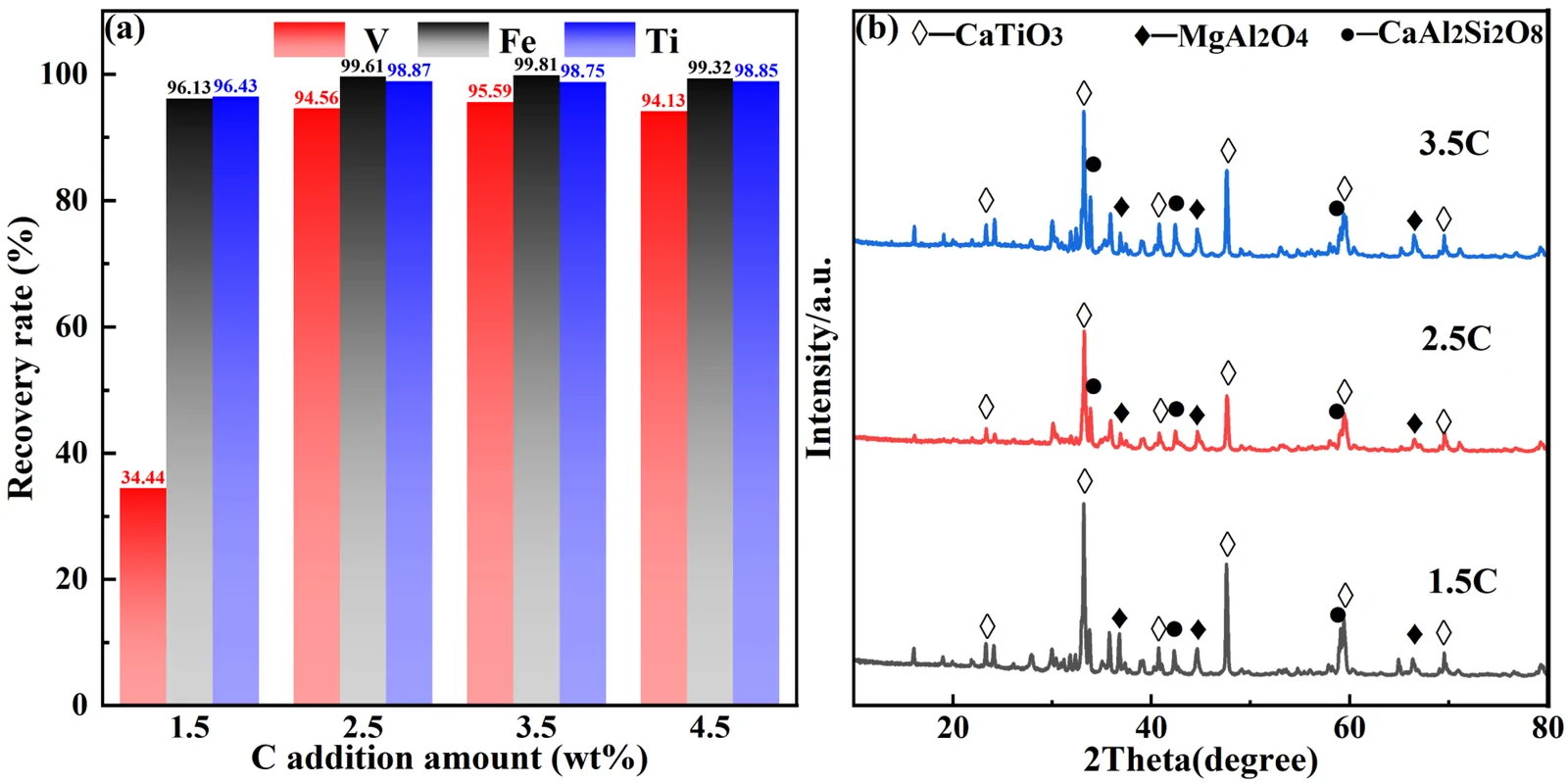
Effect of semi–coke on (**a**) the recovery of V, Fe and Ti; (**b**) XRD pattern of slag.

**Figure 20:**
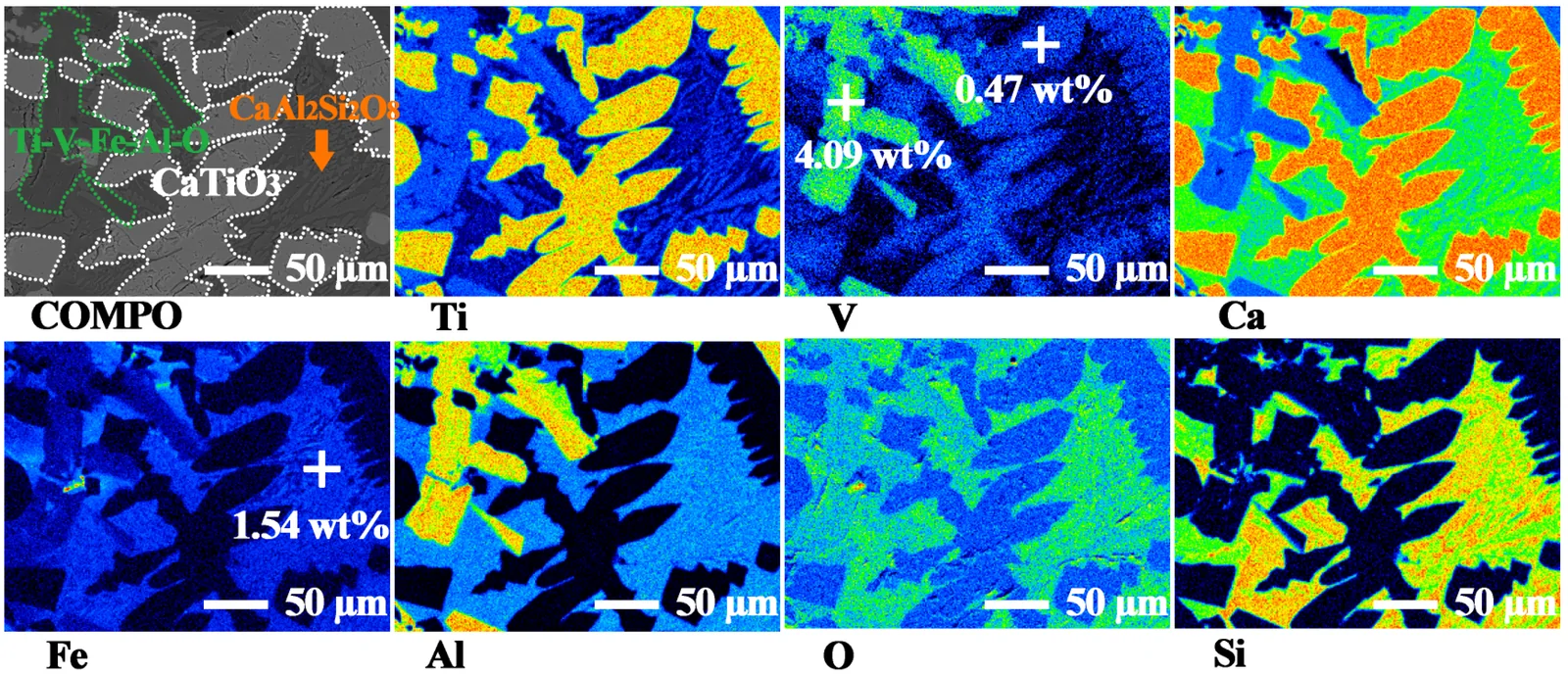
EPMA analysis of the slag with 1.5 wt% semi–coke addition.

To examine the effect of semi–coke dosage on the recovery of V, Fe and Ti from pre–reduced VTM, the pre–reduced VTM powder was mixed with 12 wt% CaO and different amounts of semi–coke (1.5, 2.5, 3.5 and 4.5 wt%), followed by pelletizing and charging into a crucible. The mixtures were then maintained at 1873 K for 20 min. At a semi–coke addition of 1.5 wt%, the residual V and Fe contents in the slag were 1.63 wt% and 1.07 wt%, respectively, and the corresponding recoveries were only 34.44% for V and 96.13% for Fe (Table 20). Compared with the 1.5 wt% semi–coke condition, the V and Fe contents in the slag decreased to 0.20 wt% and 0.23 wt% for 2.5 wt% semi–coke additions. The XRD results (Figure 19) showed that the slag at this dosage was mainly composed of CaTiO_3_, CaAl_2_Si_2_O_8_ and spinel. The EPMA results for the slag with 1.5 wt% semi–coke (Figure 20) suggested that V was mainly concentrated in the Ti–V–Fe–Al–O phase and CaTiO_3_, with contents of 4.09 wt% and 0.47 wt%, respectively. The Ti–V–Fe–Al–O phase was likely formed by segregation during slag cooling. Compared with Figure 14, the Fe content in CaAl_2_Si_2_O_8_ increased to 1.54 wt%. These results indicate that chemically dissolved Fe was not fully reduced at this stage. Combined with the low V recovery of 34.44%, this suggests that chemically dissolved V was difficult to reduce effectively by C before the complete reduction of chemically dissolved Fe, which is consistent with the thermodynamic prediction. When the semi–coke addition was increased to 4.5 wt%, the residual V and Fe contents in the slag decreased to 0.30 wt% and 0.22 wt%, respectively, while the corresponding recoveries increased to 94.13% for V and 99.32% for Fe. Under this condition, chemically dissolved Fe was nearly absent from the slag, and the V recovery increased markedly from 34.44% to 94.13%. This further demonstrated that C preferentially reduced the chemically dissolved Fe before reacting effectively with chemically dissolved V. Considering economic factors, the optimal smelting conditions for pre–reduced VTM were determined to be 1873 K with 2.5 wt% semi–coke and 12 wt% CaO.

## 5. Conclusions

The effects of MgO, CaO and semi–coke on the recovery of Fe, V and Ti were systematically investigated, and the migration and transformation pathways of V and Fe upon CaO addition were clarified.

1. With MgO addition, FeTiO_3_ was first reduced by C to V_x_Ti_3–x_O_5_ and converted to (Ti,V)_2_O_3_. MgO subsequently entered the (Ti,V)_2_O_3_ structure and displaced V, thereby promoting the release of chemically dissolved V, which was then reduced by C and transferred to the Fe–V alloy. Meanwhile, MgO was found to react with slag at 1873 K to form large amounts of MgAl_2_O_4_ (melting point: 2408 K) and Mg_2_SiO_4_ (melting point: 2163 K), markedly deteriorating slag fluidity. Slag–alloy separation was hindered, and the recoveries of V, Fe and Ti were consequently suppressed.

2. When CaO was used as the modifier, it reacted with (Ti,V)_2_O_3_ to form CaTiO_3_ and release (V_x_O_y_)_chemically dissolved V_. The chemically dissolved V was reduced to V and entered the V_Fe–V alloy_. The reaction pathway was expressed as FeTiO_3_ → V_x_Ti_3–x_O_5_, Fe → (Ti,V)_2_O_3_ → CaTiO_3_ and (V_x_O_y_)_chemically dissolved V_ → Fe–V alloy. During holding, CaO reacted with silicates to form CaAl_2_Si_2_O_8_ (melting point: 1826 K), which reduced the slag viscosity, facilitated Fe–V alloy sedimentation and improved the recoveries of Fe and V.

3. Experiments conducted at 1873 K with 12 wt% CaO and different semi–coke dosages showed that the chemically dissolved Fe was reduced by C in preference to chemically dissolved V.

4. Under the optimal conditions of 1873 K, 20 min, 2.5 wt% semi–coke and 12 wt% CaO, the recoveries of V, Fe and Ti reached 94.56%, 99.61% and 98.87%, respectively. In comparison, the conventional processes achieved Fe and V recoveries of only 90% and 70%, respectively. These results indicate that the proposed process can effectively improve the recovery of Fe and V from pre–reduced VTM, thereby enhancing energy utilization efficiency and metal yield and supporting the industrial application of pre–reduced VTM.

## Figures and Tables

**Table 1:** The chemical analysis of the pre–reduced VTM/wt%.

Element	T Fe	Fe	V_2_O_5_	TiO_2_	SiO_2_	MgO	Al_2_O_3_
Content	57.74	44.21	1.73	8.81	3.18	1.36	4.84

**Table 2:** Semi–coke proximate analysis (wt%).

Fixed Carbon	Ash	Volatile Matter
80.03	9.40	10.57

**Table 3:** Phases/species assumed and state modeling assumptions used in the multiphase equilibrium calculations.

Input Phases	Fe	Fe_3_O_4_	V	FeTiO_3_	SiO_2_	MgO	Al_2_O_3_	CaO	C
Quality	44.21	10.17	0.97	16.73	3.18	1.36	4.84	0.15	<A>
Temperature	1673 K–1873 K								
Databases	FToxid		FTmisc				FactPS		
Compound species	Ideal Gas		Pure liquids				Pure solids		
Solution phase	Fe–liq		A–Slag–liq all oxides + S				A–Spinel		

**Table 4:** Components assumed in the slag viscosity calculation.

Input Phases	SiO_2_	Al_2_O_3_	TiO_2_	MgO	CaO
Databases	Glasses				
Temperature	1673 K–1873 K				

**Table 5:** Calculated composition of the liquid Fe phase with 2 wt% C addition (wt%).

Temperature/K	Fe	V	Si	Al	C
1673	99.877	0.004	0.000	0.000	0.102
1723	99.892	0.006	0.000	0.000	0.080
1773	99.900	0.008	0.001	0.000	0.064
1823	99.903	0.010	0.001	0.000	0.052
1873	99.900	0.012	0.002	0.000	0.042

**Table 6:** Calculated slag composition with 2 wt% C addition (wt%).

Temperature/K	Fe	V	Ti	Ca	Si	Al	Mg	O
1673	20.89	3.62	19.72	0.40	5.55	9.57	3.06	37.19
1723	20.95	3.61	19.72	0.40	5.55	9.57	3.06	37.13
1773	21.05	3.61	19.71	0.40	5.55	9.56	3.06	37.07
1823	21.16	3.60	19.69	0.40	5.54	9.55	3.06	37.00
1873	21.28	3.59	19.67	0.40	5.53	9.54	3.06	36.93

**Table 7:** Calculated slag viscosity as a function of temperature with 2 wt% C addition.

Temperature/K	Viscosity/Pa·s
1673	0.100
1723	0.081
1773	0.066
1823	0.055
1873	0.046

**Table 8:** Calculated Fe liquid phase composition at 6 wt% C addition/wt%.

Temperature/K	Fe	V	Ti	Si	C
1673	94.597	1.469	0.269	0.000	3.083
1723	94.568	1.417	0.620	0.000	2.738
1773	94.581	1.374	1.074	0.001	2.345
1823	94.580	1.329	1.495	0.002	1.993
1873	94.555	1.296	1.801	0.003	1.717

**Table 9:** Calculated slag composition with 6 wt% C addition (wt%).

Temperature/K	Fe	V	Ti	Ca	Si	Al	Mg	O
1673	0.48	0.51	25.84	0.73	8.47	15.86	5.61	42.50
1723	0.47	0.64	29.49	0.65	6.70	15.48	4.96	41.61
1773	0.42	0.83	30.65	0.67	5.19	16.03	5.13	41.08
1823	0.37	1.03	31.76	0.69	3.70	16.55	5.30	40.59
1873	0.33	1.19	32.54	0.71	2.55	17.01	5.45	40.23

**Table 10:** Calculated slag viscosity as a function of temperature with 6 wt% C addition.

Temperature/K	Viscosity/Pa·s
1673	0.323
1723	0.208
1773	0.152
1823	0.120
1873	0.100

**Table 11:** Calculated composition of the liquid Fe phase as a function of CaO addition (%).

CaO/%	Fe	V	Ti	Ca	Si	C
0	94.555	1.296	0.627	0.000	1.801	1.717
1	94.515	1.392	0.631	0.000	1.608	1.849
2	94.473	1.448	0.647	0.000	1.427	2.000
3	94.417	1.484	0.693	0.000	1.236	2.165
4	94.351	1.508	0.764	0.000	1.038	2.334

**Table 12:** Calculated slag composition as a function of CaO addition (%).

CaO/%	Fe	V	Ti	Ca	Si	Al	Mg	O
0	0.33	1.19	32.54	0.71	2.55	17.01	5.45	40.23
1	0.21	0.74	30.21	5.07	3.09	15.80	5.06	39.81
2	0.15	0.49	28.10	8.84	3.52	14.72	4.72	39.46
3	0.11	0.34	26.14	12.12	3.92	13.78	4.41	39.19
4	0.08	0.24	24.33	15.00	4.30	12.94	4.14	38.97

**Table 13:** Calculated slag viscosity as a function of CaO addition at 1873 K (Pa·s).

CaO/%	Viscosity
0	0.100
1	0.076
2	0.062
3	0.054
4	0.048

**Table 14:** Calculated composition of the liquid Fe phase as a function of MgO addition (%).

MgO/%	Fe	V	Ti	Ca	Si	Al	Mg	C
0	94.555	1.296	0.626	0.000	1.801	0.003	0.000	1.717
1	94.511	1.341	0.729	0.000	1.535	0.003	0.000	1.879
2	94.472	1.380	0.819	0.000	1.281	0.003	0.000	2.043
3	94.442	1.409	0.885	0.000	1.060	0.003	0.000	2.199
4	94.421	1.431	0.926	0.000	0.874	0.003	0.000	2.342

**Table 15:** Calculated slag composition as a function of MgO addition (%).

MgO	Fe	V	Ti	Ca	Si	Al	Mg	O
0	0.33	1.19	32.54	0.71	2.55	17.01	5.45	40.23
1	0.21	0.74	30.21	5.07	3.09	15.80	5.06	39.81
2	0.24	0.73	27.37	0.61	4.02	14.66	11.60	40.77
3	0.21	0.58	25.40	0.57	4.49	13.72	14.08	40.96
4	0.17	0.48	23.75	0.54	4.79	12.90	16.26	41.10

**Table 16:** Calculated slag viscosity as a function of MgO addition at 1873 K (Pa·s).

MgO/%	Viscosity
0	0.100
1	0.080
2	0.069
3	0.064
4	0.060

**Table 17:** Effect of MgO addition on the recovery of V, Fe and Ti.

Semi–Coke/%	MgO/%	V/%	Fe/%	Ti/%	Note
2.5	3	/	/	/	Poor slag–alloy separation
2.5	6	/	/	/	
2.5	9	/	/	/	
2.5	12	/	/	/	

**Table 18:** Effect of CaO on the V and Fe contents in the slag and the Ti content in Fe–V alloy.

CaO/wt%	V in Slag/wt%	Fe in Slag/wt%	Ti in Fe–V Alloy/wt%	V in Fe–V Alloy/wt%	Fe in Fe–V Alloy/wt%	Si in Fe–V Alloy/wt%
3	1.35	0.53	0.22	0.62	98.53	0.38
6	0.58	0.26	0.36	1.27	97.15	0.35
9	0.38	0.23	0.24	1.42	97.11	0.35
12	0.20	0.23	0.16	1.57	96.99	0.33
15	0.19	0.22	0.18	1.57	97.15	0.20

**Table 19:** Effect of holding time on the V and Fe contents in the slag and the Ti content in Fe–V alloy.

Holding Time/min	V in Slag/wt%	Fe in Slag/wt%	Ti in Fe–V Alloy/wt%
2	/	/	/
4	0.55	0.46	0.5
5	0.43	0.36	0.65
6	0.33	0.28	0.6

**Table 20:** Effect of semi–coke on the V and Fe contents in the slag and the Ti content in Fe–V alloy.

Semi–Coke/wt%	V in Slag/wt%	Fe in Slag/wt%	Ti in Fe–V Alloy/wt%
1.5	1.63	1.07	0.21
2.5	0.20	0.23	0.16
3.5	0.27	0.21	0.18
4.5	0.30	0.22	0.17

## Data Availability

The original contributions presented in this study are included in the article/Supplementary Material. Further inquiries can be directed to the corresponding authors.

## Supplementary Materials

The following supporting information can be downloaded at https://www.mdpi.com/article/10.3390/ma19214582/s1. Figure S1: EPMA of alloy with 12 wt% CaO addition.
