# Supplementary material for: Transformation Behavior of Fe, V and Ti in Pre–Reduced Vanadium–Titanium Magnetite During Electric Furnace Smelting
Source: Materials (Basel). 2026 Oct 29;19(21):4582. doi: 10.3390/ma19214582 (PMC13765062; doi:10.3390/ma19214582)
Supplement: Supplementary file 1 [file materials-19-04582-s001.zip › materials-4517220-supplementary.pdf]

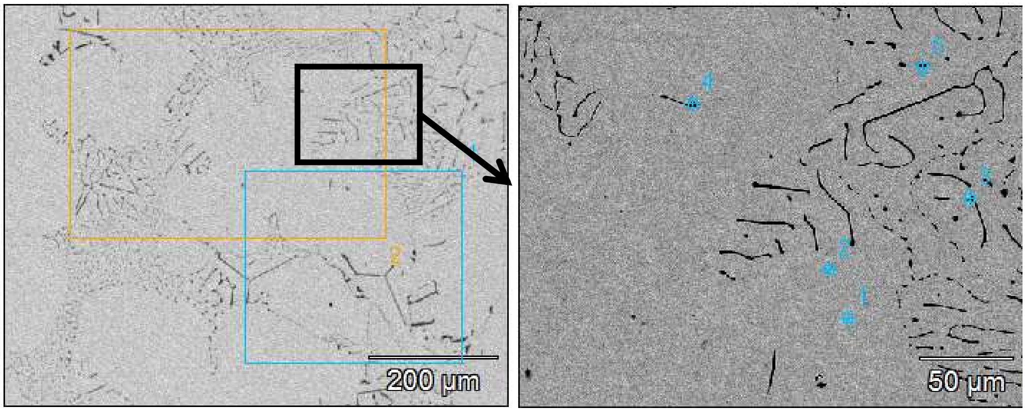

Weight%

	Si	Ti	V	Fe
Base(7881)_pt1	0.46	0.05	2.18	97.31
Base(7881)_pt2		0.00	2.22	97.78

	Si	Ti	V	Cr	Fe
Base(7882)_pt1	0.45	0.01	0.84		98.70
Base(7882)_pt2	0.37	0.05	0.81		98.77
Base(7882)_pt3	0.41	0.00	1.24		98.36
Base(7882)_pt4		7.24	83.01	1.01	8.75
Base(7882)_pt5	0.37	1.39	9.58		88.65

Figure S1. EPMA of alloy with 12 wt% CaO addition
